# The Impact of COVID-19 on the Delivery of Educational Programs in Native American Communities: Qualitative Study

**DOI:** 10.2196/32325

**Published:** 2022-04-11

**Authors:** Lea Sacca, Christine Markham, Belinda Hernandez, Ross Shegog, Melissa Peskin, Stephanie Craig Rushing, Hannah Warren, Monique Tsosie

**Affiliations:** 1 Center for Health Promotion and Disease Prevention University of Texas Health Science Center Houston Houston, TX United States; 2 Center for Health Promotion and Prevention Research University of Texas Health Science Center at San Antonio San Antonio, TX United States; 3 Northwest Portland Area Indian Health Board Portland, OR United States; 4 Alaska Native Tribal Health Consortium Anchorage, AK United States; 5 Inter Tribal Council of Arizona Phoenix, AZ United States

**Keywords:** online sexual health programs, COVID-19, COVID-19 pandemic, AI/AN youth, sexual health educators, culturally responsive adaptation, program implementation, sexual health, implementation, Native communities, American Indian youth, Alaskan youth, education, tribal communities, online, virtual

## Abstract

**Background:**

Despite the availability of culturally responsive sexual health educational programs for American Indian and Alaska Native (AI/AN) youth, barriers to their uptake and utilization persist in tribal communities. These challenges were exacerbated by the COVID-19 pandemic, which required flexible program delivery using both in-person and virtual classrooms.

**Objective:**

This exploratory study provides a preliminary understanding of the extent to which pre-existing challenges impact the delivery of culturally responsive sexual health education programs in Native communities and to what extent they were exacerbated by the COVID-19 pandemic. It also highlights the challenges faced by adolescent health advocates when adapting culturally responsive health curricula to online platforms. Finally, this study discloses major socioeconomic, health, and mental challenges experienced by AI/AN youth during the pandemic.

**Methods:**

An exploratory, descriptive, qualitative design approach was adopted to carry out 5 individual and 1 collective in-depth key informant interviews. A total of 8 Native and non-Native sexual health educators served as key informants and shared their personal experiences with the delivery of sexual health education programs for youth during the COVID-19 pandemic. The interviews were conducted virtually from October to November 2020 using Zoom to reach participants dispersed across different regions of the United States. We followed the consolidated criteria for reporting qualitative research (COREQ) as a reference for the study methodology. We also used the Braun and Clarke framework (2006) to conduct a thematic analysis.

**Results:**

Experts’ opinions were structured according to 5 main themes: (1) competing community priorities during COVID-19; (2) moving to web-based programming: skills, training, support; (3) recruiting youth; and (4) challenges for implementation in a household environment; and (5) recommendations to overcome implementation challenges. These themes are complementary, connected, and should be considered holistically for the development, dissemination, and implementation of online sexual health programs for AI/AN youth, specifically during the COVID-19 pandemic. The results raised the following points for discussion: (1) Building partnerships with schools and community organizations facilitates program adaptation and implementation, (2) there is a need to adopt a holistic approach when addressing youth sexual health in AI/AN communities, (3) a systematic and culturally responsive adaptation approach ensures effective virtual program delivery, and (4) community and youth engagement is essential for the success of virtual sexual health programs.

**Conclusions:**

Findings can provide recommendations on actions to be taken by sexual health educators and guidelines to follow to ensure cultural sensitivity, effective adaptation, and successful implementation when setting out to advocate for online sexual health programs for AI/AN youth.

## Introduction

In the United States, racial and ethnic disparities in teen births and sexually transmitted infections (STIs) persist [1]. American Indian and Alaska Native (AI/AN) females aged 15-19 years have the highest teen birth rate [1,2] and the highest prevalence of repeat teen births [3,4] compared to other racial/ethnic groups. AI/AN youth are also disproportionately affected by STIs, including HIV, gonorrhea, and chlamydia [5-7].

To address these disparities, culturally responsive programs have been receiving significant attention as a means to help public health specialists deliver culturally sensitive, evidence-based preventive practices to diverse racial/ethnic populations [8-10]. Culturally responsive programs are defined as the degree to which the cultural values, norms, beliefs, and practices of the target population are integrated into the design, delivery, and evaluation of an intervention [11]. This principle is supported, in part, by research assessing the impact of certain cultural values on several psychosocial outcomes [12]. For instance, researchers have highlighted that parental involvement in a culturally responsive, technology-based intervention to improve parent-child communication on sexual health can moderate and protect AI/AN youth from engaging in early sexual debut [13-17]. The familial system in Native culture has been consistently reported to be a protective factor for major risky behaviors among AI/AN youth [18]. Hence, efforts to address sexual health disparities among AI/AN youth through evidence-informed and culturally responsive programs are essential to improve the overall health outcomes of this underrepresented population [19,20].

Despite the availability of culturally responsive sexual health educational programs on websites, such as that of Healthy Native Youth [19], barriers remain to their uptake and utilization in tribal communities [13,21-30]. Many of these challenges were exacerbated by the COVID-19 pandemic, which required flexible delivery using both in-person and virtual classrooms [31]. Since the start of the pandemic, access to sexual and reproductive health care services for AI/AN youth has become limited in scope [22]. Native youth have also experienced challenges trying to access confidential and private sexual health information when switching to the telehealth platform, along with their inability to participate in in-person school-based sexual health education programs or attend in-person appointments in health clinics [32-36]. Maintaining youth's access to appropriate and culturally tailored sexual health education programs to increase awareness of sexual health promotion and contraceptive use is essential to prevent a peak in unintended pregnancies and STIs among this vulnerable population group [37,38].

Across the United States, many health educators relied on schools as the primary channel to deliver sexual health education to youth prior to the pandemic [39-41]. Others relied on technology-based platforms as feasible mechanisms to disseminate culturally adapted sexual health content for AI/AN youth [13,20]; enhance community expertise and resources to adopt, implement, and maintain evidence-based programs; and improve the likelihood of attaining positive sexual and reproductive health outcomes [15,21,22,24-27]. However, COVID-19 restrictions significantly affected the continuity of school-based in-person programs, since health educators were faced with the challenge of tailoring health lessons to the online platform in a short period, while ensuring the cultural sensitivity of the shared material [31]. Participation in ongoing in-person programs and recruitment for new virtual programs both were impacted by school shutdowns, particularly when trying to reach youth with limited internet connectivity or unsupportive parents in conservative areas [42].

Additionally, barriers to disseminating virtual programs in tribal communities, similar to those identified for in-person programs, continue to exist for several reasons. First, the lack of community readiness and the limited availability of resources to address sensitive topics, such as adolescent sexual health, hinders the delivery of sexual health education programs in AI/AN communities [20]. Second, inconsistencies in tribal policies, protocols, and schoolboard approval processes may generate delays in program adoption and implementation [28]. Third, poverty may result in high personnel turnover or temporary closures for AI/AN youth–serving agencies, which might negatively influence implementation fidelity and program maintenance [15]. Fourth, limited access to remote villages and rural AI/AN reservations creates a recruitment challenge for adequate program implementation [15]. Finally, competing priorities in AI/AN communities, such as food insecurity and the need to focus on other academic skills (ie, math, reading, writing) for limited virtual teaching time, might lead to a lack of support from key stakeholders. This in turn results in limited knowledge of evidence-based sexual health programs and low self-efficacy to adapt and implement them [29,30].

This exploratory study shares lessons learned by educators involved in the implementation of online sexual health programs for AI/AN youth during the pandemic. It also provides an understanding of the extent to which pre-existing challenges in the delivery of culturally responsive sexual health education programs in Native communities were exacerbated by the COVID-19 pandemic. Challenges faced by tribal health experts while adapting culturally responsive health programs to the online delivery platform are also highlighted. Finally, this study describes major socioeconomic, health, and mental challenges experienced by AI/AN youth during the pandemic. Emerging themes may assist sexual health educators in the development of key strategies for effective dissemination and implementation of virtual sexual health education programs to mitigate the impact of the COVID-19 barriers and the effects of existing underlying challenges throughout program delivery. Such guidance is of utter importance to tribal health experts who are struggling to manage new and pre-existing COVID-19 challenges influencing the successful delivery of culturally sensitive sexual health programs.

## Methods

### Study Design and Setting

An exploratory, descriptive, qualitative design approach was adopted to carry out 5 individual and 1 collective in-depth key informant interviews. A total of 8 Native and non-Native health educators served as key informants and shared their personal experiences with the delivery of sexual health education programs for youth during the COVID-19 pandemic. They also shared their diverse perspectives on the utility of using online sexual health education platforms and programs to increase reach and accessibility to youth during uncertain times. Some of the key informants were newly adapting their sexual health program to an online platform, while others were already acquainted with online delivery and had been implementing such programs for an extensive period. The interviews were conducted virtually from October to November 2020 using Zoom (Health Insurance Portability and Accountability Act of 1996 [HIPAA]-compliant Zoom session) to reach participants dispersed across different regions of the United States (Northwest, Southwest, and mid-Atlantic Pacific). We followed the consolidated criteria for reporting qualitative research (COREQ) as a reference for the study methodology (Multimedia Appendix 1).

### Ethical Considerations

The study was approved by the Committee for the Protection of Human Subjects at the University of Texas Health Science Center Houston (HSC-SPH-11-0577). The lead author obtained oral recorded informed consent from the participants prior to the start of the interview. Since the interview covered topics solely related to their professional experience, a waiver of written consent was granted, and each participant was provided, instead, with a letter of information (Multimedia Appendix 2), which described the goals and topics of the study, along with an emphasis on the voluntary nature of participation.

### Research Team

The research team comprised experts in the design and implementation of sexual health education programs for Native youth (authors CM, BH, RS, and MP) and a doctoral public health student (author LS), who served as the principal investigator of the study. LS was well trained on how to conduct qualitative research and how to effectively moderate the key informant interviews in an ethical manner.

### Participant Recruitment

In total, 15 health experts working with AI/AN youth in the United States were identified from existing publications in which they were named as authors, as well as evidence-based sexual health programs in which they were credited as principal investigators or significant collaborators. These experts had different functions in diverse fields of expertise, including adolescent sexual health, mental health, and suicide prevention. Experts were eligible to participate in the study if they (1) had experience in the adoption, implementation, and maintenance of sexual health education programs (at least 1) for AI/AN youth; (2) served tribal regions within the United States; (3) had personally experienced the impact of COVID-19 on the accessibility of sexual and reproductive health services by AI/AN youth and on the delivery of adolescent sexual health education programs; and (4) identified as Native or non-Native. Experts were invited to participate in the study by email. An attached letter of information was included in the invitation to provide additional insight into the goals and objectives of the study. Of the 15 identified experts, 8 (53%) agreed to participate. The characteristics of the participant experts are presented in Table 1. No monetary incentives were provided for participation.

**Table 1:** Expert characteristics (N=8).

Characteristics		Participants, n (%)
**Gender**		
	Female	8 (100.0)
	Male	0
**Organization type^a^**		
	Government agency	2 (25.0)
	Nonprofit organization	5 (62.5)
	Academic institution	2 (25.0)
**Occupation/role**		
	Consultant	2 (25.0)
	Project management	4 (50.0)
	Project staff	1 (12.5)
	Faculty	2 (25.0)
	Program analyst	1 (12.5)
	Evaluation coach	1 (12.5)
	Content creator	1 (12.5)
**Specialization^a^**		
	Sexual health	8 (100.0)
	Mental health	4 (50.0)
	Suicide prevention	2 (25.0)
	Youth empowerment (voting program)	1 (12.5)
**Region**		
	Northwest	4 (50.0)
	Southwest	2 (25.0)
	Mid-Atlantic	2 (25.0)

^a^Some experts work in different organizations and have multiple specializations, in addition to adolescent sexual health.

### Data Collection and Data Management

The lead author (LS) conducted the semistructured interviews via Zoom at a day/time based on the interviewees’ preferences [43]. Each interview ranged from 35 to 65 minutes. Interviews were audio-recorded and transcribed verbatim using Otter.ai software [44] after receiving the interviewees’ permission. The interview guide included open-ended questions based on 3 constructs (adoption/adaptation, implementation, and maintenance) within the broader dissemination and implementation field [43,45]. Questions prompted the interviewees on sharing challenges faced in the adoption, implementation, and maintenance of sexual health education programs for Native youth during the COVID-19 pandemic, as well as actions taken to address these challenges to implement sexual health education programs (Multimedia Appendix 2) more effectively.

The interview guide was divided into 3 main sections (Introduction, Impact of COVID-19 on the Delivery of Sexual Health Education Programs, and Use of Online Sexual Health Platforms in Response to COVID-19), with open-ended questions regarding their experience addressing COVID-19-related challenges and adapting to online delivery and recommendations for developing and implementing online sexual health programs for AI/AN youth. Most of the interviews were with individuals; however, 1 small group interview was held with 3 experts who worked together.

To protect participant confidentiality, all records were stored in locked cabinets and password-protected computer systems for use by the research team only. All (n=8, 100%) participants’ records were given a unique study ID number for data management purposes. No names were included in the data analysis files or in reports.

### Data Analysis

We used the Braun and Clarke framework [46] to conduct a thematic analysis. This framework comprises 6 phases: (1) familiarizing yourself with your data, (2) generating initial codes, (3) searching for themes, (4) reviewing themes, (5) defining and naming themes, and (6) producing the report. In the first phase, the lead author (LS) thoroughly read each transcript to get acquainted with the collected information. In the second phase, detailed descriptive coding was conducted using the comments section in Microsoft Word. In the third phase, an initial list of all codes and subthemes was generated by the lead author. In phase 4, a codebook of all potential themes and subthemes was created, including a list of definitions and quotes to support identified themes and subthemes (Multimedia Appendix 3). In phase 5, a list of candidate themes and subthemes was refined and condensed to highlight important recognizable issues and relationships between themes. In phase 6, the findings were presented in narrative form with quotes from key informants to support the identified themes and subthemes. A synthesis of the results was prepared to guide sexual health experts in the development of online sexual health programs for Native communities, while considering unprecedented challenges that might be encountered in the process.

Data analysis was conducted in a precise, consistent, and exhaustive manner by ensuring the credibility, transferability, dependability, and confirmability of our analysis, as well as the use of audit trails [47]. To check for credibility, peer debriefing was adopted to provide an external check on the research process, as well as referential adequacy, which allows for the checking of preliminary findings and interpretations of raw data [48]. Transferability was established by involving sexual health experts from diverse tribal regions to ensure generalizability of results [49]. Dependability was achieved by ensuring clear documentation and traceability of results [49]. All 3 criteria led to confirmability, along with a clear explanation of the analytical framework used in the study. Finally, keeping records of the raw data, field notes, and transcript for clear reporting of the data all contributed to an audit trail (ES Halpren, *Auditing Naturalistic Inquiries: The Development and Application of a Model*, unpublished doctoral dissertation, 1983).

## Results

### Main Themes and Subthemes

Experts’ opinions were structured according to 5 main themes: (1) competing community priorities during COVID-19; (2) moving to web-based programming: skills, training, support; (3) recruiting youth; (4) challenges for implementation in a household environment; and (5) recommendations to overcome implementation challenges. These themes are complementary, connected, and should be considered holistically for the development, dissemination, and implementation of online sexual health programs for AI/AN youth, specifically during the COVID-19 pandemic. The themes and subthemes are presented in Textbox 1 (also see Multimedia Appendix 3 for representative quotes).

**Key themes and subthemes identified in the interviews.**

**Theme 1: Competing community priorities during COVID-19**

- Food security and water sanitation measures
- Financial hardship
- Mental health impact
- Focusing on COVID-19 response in clinics and centers
- Sexual health as a secondary concern

**Theme 2: Moving to web-based programming: skills, training, support**

- Adaptation of programs to the online platform
- Lack of sufficient time and staff support in the stressful adaptation process
- Youth missing a 1-on-1 connection

**Theme 3: Recruiting youth**

- Using social media platforms for youth outreach
- Differences in the online program youth participation rate
- Distribution of flyers in community locations

**Theme 4: Challenges for implementation in a household environment**

- Challenge of youth program participation from a home environment
- Low bandwidth and network connectivity issues
- Dealing with youth internet access

**Theme 5: Recommendations to overcome implementation challenges**

- Building partnerships with schools and community organizations for program adaptation and implementation
- Adopting a holistic approach when addressing sexual health in American Indian and Alaska Native (AI/AN) communities
- Adopting a systematic and culturally responsive approach for effective virtual program delivery
- Community and youth engagement for the success of virtual sexual health programs

### Competing Community Priorities During COVID-19

#### Food Security and Water Sanitation Measures

The impact of COVID-19 on food security and water sanitation in AI/AN communities led sexual health experts to shift their roles toward food relief and building water sanitation stations. An expert highlighted the hidden food insecurity crisis in AI/AN households that is rarely mentioned in the news (Int1, where “Int” refers to “interview”). Another participant shared that there is a struggle in some households to find baby formula, which encourages AI/AN mothers to breastfeed their infants (Int2-P3, where “P” refers to “participant”). Hand-washing stations provided running water to take care of the necessary hygienic procedures during COVID-19 (Int4).

I mean, the food insecurity, I feel like we never hear about that on the news. But, um, the food insecurity is a major, major issue, like meeting the basic needs of households has been a primary thing that I've been hearing from…from Native communities and…and others, you know, that they're…they're either doing contact tracing, or they're doing food relief.

Int1

And we've also been building hand-washing stations. So, like I was saying earlier, people don't have running water. And you can ask people to wash their hands; if they don't have running water, they need to save that water to drink and to clean themselves and to, you know, cook their food. So, we've been creating hand-washing stations that we've also been delivering to our various partners.

Int4

#### Financial Hardship

Of the 8 experts, 4 (50%) highlighted the financial hardship AI/AN families are struggling with as a result of the COVID-19 pandemic (Int1, Int4-Int6); 1 (25%) of them expressed the financial burden caused by COVID-19 on AI/AN parents and youth because youth had to exchange the opportunity to participate in a sexual health program with the need to find a job to support their families (Int1). A participant also reflected on the long-standing economic inequities that became more apparent during the pandemic (Int4).

They have to deal with food insecurity and, like, unemployment, so the kids have to help their parents; that is a national dilemma, right, you know…everybody sort of, kind of everything sort of falling away. But the basics, you know…and you know, it's an unfortunate thing about this pandemic, is it's really just shedding a light on long-standing inequities and deep-seated inequities between groups in this country. And you know, it's really just shining light on that. And hopefully, some good will come of it.

Int4

#### Mental Health Impact

Many participants expressed concerns regarding youth’s mental health during the pandemic. Based on her experience, 1 (12.5%) expert described the detrimental mental health impact of COVID-19 on youth due to the stress, anxiety, and worries associated with the unknown duration of the pandemic and the severity of the disease (Int2-P2). The lack of stability and the loss of elder lives were devastating at a community level (Int3, Int4). An additional concern was having youth feeling “zoom fatigue” as they also complete their schoolwork online (Int3). An expert emphasized the need to support youth since even though they seem to be handling the pandemic well, a spike in mental health conditions is emerging among this population (Int5). Another warned about the trends in youth mental health impacts over the next decade (Int6).

And I think, we're at real risk of, like, a widespread mental health crisis for young people, for teens, particularly. So, we cannot…we cannot not address that, like young people's lives are at stake when it when it comes to a time like this. So that would be like mental health piece is 1 of…my number 1 thing to do would be to build competence in how to do this and be really, really thoughtful about how you plan and prepare and deliver your programming.

Int1

And we've all kind of heard this; like zoom fatigue is such a thing where you're just on video calls all day, and you're exhausted. So, we didn't want this to be 1 more thing that a kid has to do that it's something that they want to do.

Int3

#### Focusing on COVID-19 Responses in Clinics and Centers

A common subtheme across all interviews was the shift in clinic focus away from providing core public health services toward COVID-19-related relief efforts. A participant explained how their Tribal Epidemiology Center pivoted to deal with the COVID-19-related testing and contact tracing, whereby all project-related staff were helping manage the COVID-19 surge in AI/AN communities rather than focusing on youth sexual health programs (Int3). Hesitancy to seek sexual health care during the COVID-19 pandemic was pointed out by an expert in the Northwest, as people did not want to increase their risk of exposure in small clinic spaces for regular checkups (Int2-P3). An expert shared about the switch to telehealth platforms to increase youth access to sexual health programs; however, accessibility differed across tribes (Int6).

And through our partnerships with [the Indian Health Service] (IHS), we've also been asked to help them with other COVID-related efforts. So, some of that, but all that work is new, and not necessarily what our team was doing prior to COVID. And it's constantly changing, and in flux…that both of the communities that we work with the most closely, there have been weeks where we have our team back, and it's almost as if, you know, it's not COVID, staff attendance, and availability, but then, you know, the surge…and we lose some of our staff members to necessary COVID-related work.

Int3

#### Sexual Health as a Secondary Concern

Of the 8 experts, 5 (62.5%) reported that sexual health moved from a community-wide health priority to a secondary concern due to challenges imposed by COVID-19 at the mental, economic, and nutritional levels. In addition, 1 (12.5%) participant expressed the need for a holistic approach to sexual health as people are prioritizing their basic needs and ensuring that all their family members are safe (Int2-P2). In terms of prioritizing needs, an expert emphasized the importance of relationship building by reframing messages in an informal context (Int6).

And now we're seeing a federal response saying here is a curriculum, a list that can be utilized for some of these programs. I think what's interesting is that it's just not the silo of physical sexual health. It's more holistic—mental health, social health, cultural health, and physical health.

Int2-P2

### Moving to Web-Based Programming: Skills, Training, Support

Since the start of the COVID-19 pandemic, the majority of in-person and hybrid sexual health education programs had to be adapted to online learning platforms. Experts discussed major challenges faced in the delivery of virtual programs from their own perspective and from the youth's and communities' perspectives. The challenges were most apparent among experts adapting and translating health education programs to virtual platforms for the first time (Int3-Int5).

#### Adaptation of Programs to the Online Platform

Of the 8 participants, 1 (12.5%) described the challenge of trying to figure out how to coordinate with schools to plan and deliver virtual sexual health programs for youth while managing their ongoing hybrid learning platform (Int1). Other experts expressed struggling to identify which program components to keep, since most activities were designed for in-person delivery (Int3, Int4). A concern shared by most experts was ensuring that the adaptation process was not completed hastily to ensure they met youth’s needs (Int1-Int4). In addition, 2 (25%) experts indicated that the in-person sessions were too long when adapted to the virtual platform and had to be shortened to ensure youth engagement throughout the session (Int3, Int4). Furthermore, 1 (12.5%) expert shared that a lot of adaptations needed to be made even for programs that used the online platform for content delivery, since these programs had a physical component to some extent prior to the COVID-19 pandemic (Int3).

It can be very quick to open up a Zoom account and get started on Zoom, right? You can do it in minutes. But that doesn't mean that you're prepared to implement a program online. So, I think that 1 of the challenges is that people are going to, I think, initially hastily put their programs together, and they're not going to be good. And they're not going to actually meet the needs of young people.

Int1

You know, very quickly, we had to start adopting our own programming for virtual settings, and, you know, kind of identify which components of our work we're going to get [to] continue happening in community settings, because most of our tribal clinics closed, our tribal schools closed, public schools closed. So, kind of being in touch with our tribal health educators and our clinic staff to see which services and programs were continuing, what was being paused, what priorities they had for moving forward and trying to respond to those.

Int2-P1

#### Lack of Sufficient Time and Staff Support in the Stressful Adaptation Process

Experts described the adaptation process as stressful for staff due to the limited time frame available. Of the 8 experts, 1 (12.5%) disclosed that staff were begging for support because meeting young people's needs was substantial during the pandemic. Another highlighted the gap in resources provided for employees working from home (Int5). In addition, 2 (25%) experts from the collective interview (Int2) described the process as “trial and error” due to the time constraints tribal employees encountered while becoming “Zoom savvy” for virtual programs. A common subtheme shared by all experts was the need for continuous staff training to get acquainted with the online platforms and software available to maximize youth learning and engagement. Participants believed that professional development is needed to help staff feel equipped to navigate virtual platforms (Google Classroom, Zoom, Jamboard, etc; Int3).

Secondly was how to coordinate our schedules and utilize a virtual platform such as Zoom and become Zoom savvy for us as professionals, and I can tell you, I think we've talked about it, we've been more busy now in a virtual setting than we have when we were in the office.

Int2-P2

So, although some of these were presented in person, there was a lot of adaptation to go online, and what that looked like, so a lot of trial and error, a lot of challenges, and a lot of different avenues of communication and having to be also creative and also still innovative, too.

Int2-P3

We had to incorporate an entire tech training on how to use Zoom and how to use, like, the internet to do a lot of things. And I mentioned our team, our paraprofessionals, which means they're from the community, but they may not always have all of the skills. So, for us, training is really important to make sure that we're helping our team members reach their best potential so they can do all of these things.

Int3

#### Youth Missing a 1-on-1 Connection

Of the 8 experts, 4 (50%; Int3-Int6) emphasized that youth were missing the 1-on-1 connection established in in-person programs. Therefore, experts had to delete or modify online activities to engage youth as much as possible. The in-person component was pointed out as an integral part of the AI/AN culture (Int6).

We have had some Zoom sessions where there are, you know, siblings together with their parent, and there's a couple kids, but their original program, you know, kids are in groups, and they're doing all these fun activities, and they're laughing, and they're really engaged. It's very much like an interactive process. And so, you know, some of the activities that were like that originally—that we knew we couldn't do on Zoom—we either took out or modified.

Int4

But for tribal communities, the in-person part is such a major piece. And I don't see that changing in any capacity. I do think that the virtual piece will be really amped up, and I think it will be smoothed over quite a bit [of time].

Int6

### Recruiting and Retaining Youth

#### Using Social Media Platforms for Youth Outreach

Based on the 2020 nationwide Youth Health Tech Survey [50] mentioned by 1 (12.5%) of the 8 participants, certain social media platforms were identified as preferred channels by AI/AN youth to receive sexual health messages and lessons (Int6). A participant pointed out that facilitators used their personal social media to reach out to youth and families in their community. One expert also mentioned that social media facilitated recruitment during the pandemic, since social messaging was popular among youth (Int2-P2). Another expert emphasized the effective role of social media in allowing lesbian, gay, bisexual, transgender, and questioning (LGBTQ) youth to open up about their sexual health and body image in a positive manner due to the support received from the online platform (Int2-P3). All experts stated that TikTok is less often used to reach youth than are Instagram, Facebook, YouTube, and Twitter. We R Native, a multimedia health resource for Native youth, has a large and growing presence on social media. Instagram and Facebook were the main channels used by We R Native to promote sexual health resources during the pandemic, due to their popularity and national reach. Since then, they have seen expansive growth on TikTok, too—addressing healthy relationships, condoms, STI testing, and birth control with an “indigenous” lens.

#### Differences in the Online Program Youth Participation Rate

Recruitment and retention of youth for online sexual health program participation was reported as problematic by 2 (25%) of 8 experts (Int5, Int6). Most health educators relied on schools to locate participants and increase recruitment and retention rates (Int5). However, since the start of the pandemic, schools were shut down, and more youth sought mental health services rather than focusing on their sexual health (Int6). A participant described the negative impact of school shutdown due to COVID-19 on youth recruitment for online sexual health programs. Schools were considered a major hub for recruitment and created an organized structure for outreach efforts (Int1). Parents are also more comfortable when their youth participate in school-based curricula rather than having an outside organization deliver the program (Int1). Most AI/AN villages and reservations are considered remote, which renders schools a hub for recruitment purposes (Int3).

I think the programs that are going to be successful at recruitment is programs that are operating through a school. It's much harder already; like some of my teams in the Midwest have been trying to get the young people together, and they're just now getting to the point. And it's because school has created this, like, sort of organizing opportunity, like kids are having a more structured day than what they have over the summer. And in the early days of the shutdown.

Int1

Yeah, there’s definitely…the recruitment and the retention in a virtual setting…versus an in-person implementation. Yes, there is a dramatic difference. Only because of the fact that, you know, that we're trying ourselves as an organization, and educators trying to find resources and tools that they can implement, if they were to implement a [curriculum], trying to adapt that. The upside that we're seeing is an uptick in our social messaging for youth. That's a very popular resource.

Int2-P2

#### Distribution of Flyers in Community Locations

Prior to the COVID-19 pandemic, in-person recruitment was highlighted as 1 of the most effective ways to reach out to AI/AN communities and inform them about the program. A participant indicated that there still needs to be an in-person component to ensure high engagement and excitement about the program despite the restrictions imposed by the pandemic (Int3). Operating through channels available in the community facilitated getting the word out about programs and overcame the challenge of limited network connection and bandwidth (Int1).

That's 1 of the effective ways. I mean, flyers in a place that people are still going, in some of the smaller Native communities. There may be people [who] actually go and visit a bulletin board, or they go and visit, like, something in a primary building. Or especially, where the food pickups are happening…a flyer on a bulletin board or a flyer in a location like that can be a good tool for getting the word out.

Int1

### Challenges for Implementation in a Household Environment

#### Challenge of Youth Program Participation from a Home Environment

Another challenge discussed by experts (Int3-Int6) was youth’s compromised participation in online sexual health programs from their households due to conflicting parent schedules, sharing equipment with siblings, and being surrounded by family members when learning about sexual health. Unreliable internet connections and the limited access to computers in rural AI/AN households have been described by 1 expert as an ongoing “digital divide” (Int2-P2). Many youths do not have access to computers at home or have to share their laptops with their entire family, which makes it harder to participate in virtual sexual health programs (Int3, Int5, Int6). Additionally, 1 of the main program delivery challenges discussed by these experts was having youth in unsafe households (physical and sexual abuse) to participate in such programs, as well as parents perceiving sex as a taboo topic. Delivering these programs through schools helped overcome these barriers, because health educators did not have to worry about conservative parents monitoring the programs' content. As a participant pointed out, even in virtual clinical visits, health care providers are asking whether youth are in a safe place to discuss their health concerns (Int1). Some programs are even including parents in sessions to increase parent-youth communication and appease their fears about having their kids participate in online sexual health programs (Int2-P2, Int3, Int5).

But you know, there's lots of need around, you know, with intergenerational families sharing technology. So, you know, maybe the youth was assigned a computer that or, you know, a tablet that did have data on it, but then that youth might have to be sharing it with siblings or the adults. And then also family schedules. So, you know, if there's a family and this youth is supposed to be on a sexual health lesson, but then mom needs to go grocery shopping, just take all the kids with her.

Int6

So, for instance, young people that are living in homes that aren't safe, and that may be like participating in a sexual health program, in a household where that is not accepted. Whereas a young person could have previously like said, “Oh, my after-school program is, like, [a] spirit club or something,” when it was actually a sexual health program. Um, you know, now, if they're doing something at home, they are in a home where other people may see what they're doing and know what they're up to. And in homes that are not safe to do that, that can be a real barrier.

Int1

But we've also heard collectively what the needs are. And…and that's pretty compelling from the field, infrastructure, needs. You know, there is a digital divide out there. So, not everybody can utilize a computer and a laptop or enroll America in most places. So, that's been pretty compelling.

Int2-P2

#### Low Bandwidth and Network Connectivity Issues

One of the main challenges encountered by experts in the delivery of online sexual health programs is the low bandwidth in rural households and network connectivity issues in tribal communities. It is thus important to be mindful of youth's network connection when designing program activities, because it might take youth some time to connect with their facilitators and ensure a stable internet connection without dropping out of sessions (Int3, Int4). Most youth have reported adequate access to technology resources. However, an expert pointed out the need to continuously provide technical support for tribes with varying capacity and resources to ensure engagement in virtual programs (Int6).

Yeah, I think overall, we know that youth are able to access and have technology resources. And so, if that's the case, then this is the best platform to do it. It's a pretty small number of folks who have absolutely no IT capacity whatsoever; it seems like most tribes, at least, you know, can lend a laptop and a thumb drive. And it's not a perfect scenario. But that's an adaptation that folks are making.

Int6

#### Dealing with Youth Internet Access

Building partnerships with schools was described by a participant as the gold standard due to tribal preference of working with schools to reach youth in remote areas through a streamlined process (Int6). Schools can help resolve the issue of limited internet connection and bandwidth that youth have in their households, which can affect program access and completion (Int4, Int5).

And then, at least in schools, they can still access the program and complete everything, and they don't have to worry about [the] internet connection or, like, the bandwidth.

Int4

### Recommendations to Overcome Delivery Challenges

#### Building Partnerships With Schools and Community Organizations

All (n=8, 100%) participants emphasized the necessity of building partnerships with schools and community organizations as essential for online program adaptation and implementation during the COVID-19 pandemic. Prior to the pandemic, schools were considered a major hub for youth recruitment and facilitated youth’s access to adequate internet bandwidth and computers to participate in virtual sexual health programs [51,52]. However, the pandemic forced health educators to think of alternative ways for maintaining program delivery due to school shutdowns and restrictions on in-person activities [53]. This in turn led educators to adapt lessons for in-person and hybrid delivery [53]. Experts shared the need to collaborate with schools when preparing to transition to online platforms. Partnerships can also facilitate the provision of continuous staff support and training to simplify the adaptation process.

You know, parents often do trust what's happening at school a lot more than they trust an outside organization; they're a lot more suspicious of even things like a Boys and Girls Club, you know. So well established, like doing a social good in the world. So yeah, I do think that working in partnership with a school or another bigger organization that can give you a little bit of cover and decrease the suspicion now, you know, the alternative may be true in some Native communities if the institution is a non-Native institution, because there's a lot more suspicion around those types of organizations [than] there might be in White communities, so things like a university [are] not necessarily a great implementation partner in some Native communities that have suspicion around, you know, universities that have historically done research on them.

Int1

There are collaborations happening between, for example, health, community health, [and] behavioral health departments within a health setting, partnering with their local school, you know, their PE or health teacher, their program, and you know, so you have 2 entities coming together to offer this class. So, that's becoming very popular; it can be done for credit recovery…it could be done just for the sheer fact that this is a tribal school on tribal land that's looking for tribal self, sexual health. Very, very popular. It's the trend.

Int2-P2

I think that's always the gold standard, too, is that most tribes want to…most programming want to work with schools, because that's the easier way to get to youth and it's a more organized and streamlined process. So, I think that will always be the goal—to collaborate with schools.

Int 6

#### Adopting a Holistic Approach When Addressing Sexual Health in AI/AN Communities

Addressing sexual health holistically was 1 of the major themes emphasized by the interviewed experts in this study. For years, sexual health educators have been emphasizing the need to have a holistic approach to sexual health interventions as STI/HIV prevention interventions were not inclusive of other important adolescent health issues [54-56]. Social and emotional learning, along with physical and mental health issues, must also be addressed in these interventions to promote positive youth development [56]. Since the start the COVID-19 pandemic, downward trends in mental health became apparent in AI/AN communities, due to parental unemployment, food insecurity, and home-based learning with limited social connection [57].

If your tribe has behavioral health services, or mental health services, that can support you in integrating those topics into your sexual health program, I think that this is the time to do it, because young people are struggling. And I think our we're at [a] real risk of, like, a widespread mental health crisis for young people, for teens, particularly.

Int1

#### Adopting a Systematic and Culturally Responsive Approach for Effective Virtual Program Delivery

Experts disclosed the benefits of adopting a systematic approach for effective implementation of online programs during the pandemic. Their recommendations included making a leap to identify what is working in AI/AN communities, rather than getting paralyzed just thinking it through; knowing which specific tools are relevant for the program rather than using all available tools and websites; and being patient since program adaptation is a strenuous learning process, particularly during the pandemic, where things are changing all the time. An additional recommendation was the need to have sexual health programs that are reflective and inclusive of the health belief systems of Native cultures and Native traditions since there is so much to learn from Native cultures when it comes to health and well-being.

I think another tip to a part of that was just like, you know, technology can be big and intimidating. I heard a lot of people say that—multigenerational, from the youth all the way to adults. And so, I think it's…it's okay if there's patience, you know; technology's not going anywhere. Even if they feel that they want to get a good grasp of what that looks like, or how to do it. I think it's nice to just kind of get your bearings and then proceed on.

Int2-P3

I guess some general advice would be to just make the leap. I think sometimes, you get paralyzed by thinking it through. But you just have to act and try to see what's going to work and know that have a flexible approach that you can change things around if things aren't working.

Int3

I think that there's so much to be learned from Native cultures when it comes to health and well-being. And so, ensuring that programs are inclusive and reflective of that and are rooted in the culture and the community itself [is] really important, really essential. You're not going to get a program off the ground without those things.

Int4

#### Community and Youth Engagement for the Success of Virtual Sexual Health Programs

Interviewed experts emphasized the need to continuously engage youth and community members, to listen to their feedback and carry out the suggested improvements, while considering the impact induced by the pandemic on their overall well-being. Focus groups with program participants and community members can improve program sustainability by highlighting the COVID-19-related challenges that need to be addressed.

And what can be helpful for the youth as well as for the adult audience to know, the 1 thing that resonates across the board is—Natives like Natives, you know—we want to see ourselves in the products in the curriculum, in the videos in the messaging, and [in] the theme. And if you tie it back to culture, then that's the tagline—culture is prevention.

Int2-P2

I think the first thing is, you have to have real community engagement, and willingness and belief that the program is important, and that the program needs to come from the community itself.

Int4

## Discussion

### Principal Findings

This study aimed to (1) better understand the extent to which pre-existing challenges were exacerbated by COVID-19, (2) examine barriers encountered when adapting programs to an online environment, and (3) highlight socioeconomic challenges experienced by youth. Both experts who had extensive experience adapting and translating health programs to online platforms and experts who were going through the process for the first time were interviewed. Such diversity in perspectives allows for a broader exploration of the impact of COVID-19 on the adoption, implementation, and maintenance of youth sexual health programs across different tribal regions in the United States. One of the strengths of this paper is that experts did not restrict themselves to sharing their professional point of view. Rather, they shared the experience of participating in online health programs from the perspectives of youth, parents, and families in tribal communities. Further, they provided advice and recommendations for future sexual health programming, with flexible options for program delivery.

As described by the key informants, AI/AN youth experienced significant and prolonged disruptions to sexual health education and sexual health services during the pandemic. Many also experienced socioeconomic and mental health challenges, juggling virtual learning while supporting their family’s basic needs. There is a need for cross-agency funding opportunities that holistically support the health and development of AI/AN youth [56,58-62]. Many tribes are small, which makes it challenging to apply for issue-specific funding. Holistic funding opportunities will provide tribal health educators with the opportunity to address the social determinants of health and the risk factors leading to adverse health outcomes among AI/AN youth [56].

To better disseminate culturally responsive resources for future program adopters, experts recommended Healthy Native Youth [63], We R Native [64], and iknowmine [65] websites that share resources, tools, and curricula to get people engaged and excited about topics, ideas, and strategies that communities have used to address AI/AN youth sexual health. Even though technological tools can be intimidating at a multigenerational level (youth and adults), experts stressed the importance of being patient with technology, as well as the need for facilitators to have a good grasp of technological tools before moving on with program adoption or implementation.

Collaborative partnerships between AI/AN communities have been reported as an effective strategy to improve program delivery [50]. Successful programs implemented in AI/AN communities have been attributed to all the connections made across different project partners and collaborators [51]. One expert shared that all things are rooted in relationships in Native communities. Another expert emphasized the importance of attending meet-and-greet sessions to learn from the personal experiences of program implementers working in the field. A common recommendation highlighted across interviews was the power gained from leveraging community-mobilizing efforts during the pandemic, along with believing and trusting in the project staff who bring their strengths and talents to the table. As mentioned by 1 (12.5%) of the 8 experts, even though COVID-19 has altered the way sexual health programs are being delivered to youth, patience and perseverance will create the needed answers in these uncertain times.

Findings from this study reiterated the importance of community and youth engagement in the dissemination, adaptation, and evaluation of health promotion programming in Native communities. In a systematic review looking at the elements of a successful implementation framework in indigenous communities, two-thirds of included studies demonstrated high levels of community engagement from a culture-centered approach, while two-thirds of the studies included structural changes and researcher reflexivity [58]. Similarly, a review of effective youth engagement strategies for mental health and substance use interventions indicated that comment boxes and evaluation surveys as well as primary decision-making authority at every stage of program design, implementation, and evaluation contribute to high youth engagement [59]. Other strategies for youth engagement include having youth sit on boards and committees within an organization and having youth participate as peer support workers [60]. Findings from the included studies emphasized that youth participation in program adaptation established a dissonance between their behavior in using substances and their prevention role adopted through program participation [61]. Additionally, youth participants who were able to better identify with the program content recorded a significant reduction in adverse health behaviors [62].

### Limitations

This study has several limitations. Given that participant recruitment was conducted using a nonrandom sampling approach, selection bias cannot be ruled out. However, the purposive sampling approach allowed us to reach a diverse group of sexual health experts across different US regions. The small sample size is attributed to the low response rate to recruitment emails and the hard-to-reach sexual health experts who were likely overburdened with the challenges in program delivery imposed by COVID-19. This is an inherent limitation of the recruitment methodology that was addressed through the detailed descriptions, thoughts, and themes provided by the key informants, along with the diverse demographic characteristics of the sample. Furthermore, the diversity of experts interviewed made it possible to obtain opinions from organizational, field, and academic professionals. Finally, another limitation was having 1 collective key informant interview encompassing 3 key informants who might have influenced each other’s opinions; yet the allocation of questions for each participant helped control for any kind of potential biases.

### Conclusion

This exploratory, qualitative study examined COVID-19-related challenges in the adaptation and delivery of sexual health programs on virtual platforms. Recommendations for future efforts included building partnerships with schools and community organizations, adopting a holistic approach to sexual health in AI/AN communities, adopting culturally responsive approaches, and engaging youth and community members in the design and delivery of sexual health programs in AI/AN communities. Findings can provide guidance on strategies to follow when selecting or preparing online sexual health programs for AI/AN youth. Future studies should explore the impact of COVID-19 on sexual health programs from the perspectives of youth themselves and empower them to share their own thoughts and recommendations for effective sexual health programs when delivered in hybrid and virtual spaces.

## Appendix

Multimedia Appendix 1 COREQ checklist. COREQ: consolidated criteria for reporting qualitative research.

Multimedia Appendix 2 Letter of information, interview guide, codebook, and theme generation.

Multimedia Appendix 3 Quotations for themes and their relevant subthemes.

## Abbreviations

AI/AN: American Indian and Alaska Native
STI: sexually transmitted infection
