# Supplementary material for: The Impact of COVID-19 on the Delivery of Educational Programs in Native American Communities: Qualitative Study
Source: JMIR Form Res. 2022 Apr 11;6(4):e32325. doi: 10.2196/32325 (PMC9004623; doi:10.2196/32325)
Supplement: Multimedia Appendix 1 [file formative_v6i4e32325_app1.docx]

APPENDIX 1. COREQ CHECKLIST

| **Domain 1 : Research team and reflexivity** | **Personal characteristics** | |
| --- | --- | --- |
|  | 1. Which author(s) conducted the interview? | Interviews conducted by LS |
|  | 2. What were the researcher’s credentials? | LS: PhD candidate in public health; CM, BH, MP, and RS: PhD |
|  | 3. What was their occupation at the time of the study? | LS: PhD candidate in public health; CM, RS, & MP: Professor in Health Promotion; BH: Assistant Professor in Health  Promotion |
|  | 4. Was the researcher male or female? | 4 females, 1 male |
|  | 5. What experience or training did the researcher have | Experience in conducting qualitative research (all authors), experience in interventional research surveys (all authors), expertise in public health (all  authors), expertise in sexual health (CM, BH, MP, and RS) |
|  | **Relationship with participants** | |
|  | 6. Was a relationship established prior to study commencement | The interviewer did not know most of the participants before the study. Three professionals were colleagues by training and were collaborating on a grant with the authors of the study. |
|  | 7. What did the participants know about the researcher? | At the start of the study, the aim of the research project, as well as the objectives of the study was presented. |
|  | 8. What characteristics were reported about the  interviewer/facilitator? | Interviewer characteristics were not reported to participants |
| **Domain 2: Study design** | **Theoretical framework** | |
|  | 9. What methodological orientation was stated to underpin  the study? | We used thematic analysis in a sociological theoretical approach. |
|  | **Participant selection** | |
|  | 10. How were the participants selected? | Participants were recruited for their experience in sexual health, prevention and health promotion, youth health; program development, implementation, and maintenance; in direct contact with youth, at the level of research and institutions.  We tried to represent a diversity of profiles  and backgrounds. |
|  | 11. How were the participants approached? | Originally by email |

|  | 12. How many participants were in the study? | 8 |
| --- | --- | --- |
|  | 13. How many participants refused to participate or dropped out? Why? | 7 (overwhelmed with COVID-19 relief efforts and program adaptation to the virtual platform) |
|  | **Setting** | |
|  | 14. Where was the data collected? | On Zoom (HIPAA compliant) |
|  | 15. Was anyone else present  besides the participants and researcher? | No. One interview involved three participants. |
|  | 16. What are the important characteristics of the sample? | Diversity of backgrounds and occupation (see characteristics in table 1) |
|  | **Data collection** | |
|  | 17. Were questions, prompts, guides provided by the author? Was it pilot tested? | The interview guide was tested, read and adapted during the interview according to the expertise of each participant. |
|  | 18. Were repeat interviews carried out? Details | No repeat interviews. |
|  | 19. Did the researcher use audio or visual recording to collect the data? | All interviews recorded. |
|  | 20. Were field notes made during and/or after the interview or focus group? | Notes taken during all interviews. |
|  | 21. What was the duration of interviews or focus groups? | From 35-65 minutes |
|  | 22. Was data saturation discussed? | Data saturation was discussed after the fourth interview. |
|  | 23. Were transcripts returned to participants for comments and/or correction? | Transcripts not returned to participants |
| **Domain 3: Analysis and findings** | **Data analysis** | |
|  | 24. How many data coders coded the data? | One author (LS) created the initial coding tree using first samples interview. The  coding process was reviewed by all authors. |
|  | 25. Did authors provide a description of the coding tree? | The coding tree is the one presented in Table 2 and corresponds to the themes and sub-themes identified. |
|  | 26. Were themes identified in advance or derived from the  data? | The themes were derived both inductively and deductively |
|  | 27. What software, if applicable, was used to manage the data? | None |

|  | 28. Did participants provide feedback on the findings? | No feedback was obtained from participants |
| --- | --- | --- |
|  | **Reporting** | |
|  | 29. Were participant quotations presented to illustrate the themes/findings? Was each quotation identified? | We present some quotations to illustrate findings. |
|  | 30. Was there consistency between the data presented and the findings | The data presented and the findings are consistent |
|  | 31. Were major themes clearly presented in the findings? | We present the most important themes related to the study objectives in the findings |
|  | 32. Is there a description of  diverse cases or discussion of minor themes? | We report and describe diverse cases |
