# Supplementary material for: The Impact of COVID-19 on the Delivery of Educational Programs in Native American Communities: Qualitative Study
Source: JMIR Form Res. 2022 Apr 11;6(4):e32325. doi: 10.2196/32325 (PMC9004623; doi:10.2196/32325)
Supplement: Multimedia Appendix 2 [file formative_v6i4e32325_app2.docx]

**Multimedia Appendix 2. Letter of Information**

**The Impact of COVID-19 on the Delivery of Sexual Health Programs in Native Communities**

Dear Key Informant,

American Indian and Alaska Native (AI/AN) youth experience serious disparities in sexual and reproductive health. These disparities may be reduced by implementing sexual health education programs in these communities. Although a growing number of evidence-based programs have been developed or adapted for Native Youth, many factors may hinder their adoption and implementation in Native communities. One of these unprecedented factors that has severely impacted the adoption, implementation, and/or maintenance of such programs is the COVID-19 pandemic.

To help us with understanding the extent of the consequences generated by COVID-19 on sexual health education programs in Native communities, we are conducting key informant interviews with experienced individuals who have witnessed the impact of the pandemic on the implementation of sexual health programs for Native Youth. We would like to hear what was your overall experience in delivering programs during the pandemic, what were the main challenges and dissemination and implementation barriers you faced, what were the main challenges for youth when engaging in these programs, what additional resources were needed, how the online platform was affected by COVID-19, and finally how you addressed these challenges and/or how you plan to address upcoming obstacles in the midst of the ongoing pandemic or with future similar disasters.

We would like to ask you to participate in one key-informant interview, which will last approximately 45 minutes. The program coordinator for the project will virtually set up a meeting via Zoom at a convenient time and will administer the interview to you. The interview is completely voluntary. You can withdraw from the interview at any time, and you can refuse to answer any questions you do not wish to answer. Your employment at your organization will not be affected in any way if you choose not to participate in the interview. The information you provide is confidential. You will not be personally identified in any reports or publications that may result from this study.

Your participation in this interview will help us to better understand the impact of COVID-19 on the process of adopting and implementing sexual health education programs in Native communities. It will also provide us with a new perspective regarding adapting and creating programs using multiple online platforms for Native Youth. There are no known physical risks in taking part in this study. However, some individuals may feel some discomfort in answering sensitive questions about sexual health education programming.

There is no cost to you for joining this study.

Thank you very much. If you have any questions, please contact Christine Markham, Principal Investigator (713-500-9646).

Sincerely,

Christine Markham, Principal Investigator University of Texas Houston Health Science Center, School of Public Health

**CPHS STATEMENT: (HSC-SPH-18-0958) This study has been reviewed by the Committee for the Protection of Human Subjects (CPHS) of the University of Texas Health Science Center at Houston. For any questions about research subject’s rights, or to report a research-related injury, call the CPHS at (713) 500-7943.**

**Key Informant Interview Guide**

Thank you for agreeing to participate in this interview. We want to hear about your overall experience with how you went about providing sexual health education in your community during the COVID-19 pandemic, what helped, the challenges you encountered, and how you managed them. We also want to hear about your perspective on adapting or creating new sexual health education programs using the online platform. Great, Let’s get started!

**Introduction**

Which adolescent sexual health education program(s) have you used in your community? What was your primary role(s)?

**Impact of COVID-19 on the Delivery of Sexual Health Programs**

1. Can you please start by describing your overall experience with COVID-19 in your community, particularly in your field of work?
2. What were the main consequences caused by COVID-19 on ongoing sexual health education programs in Native communities?
3. What are the top challenges and barriers affecting your ability to provide sexual health programs to youth in your community?
4. How has the COVID-19 pandemic impacted youth’s ability to access sexual health programs?
5. To what extent have youth been affected in accessing reproductive healthcare services in your community during the pandemic?
6. What were the actions taken by community leaders and sexual health experts to address the current COVID-19 related challenges?
7. How will you be planning for the delivery of future sexual health programs in the midst of the ongoing COVID-19 pandemic?

**Online Sexual Health Platforms to Deal with COVID-19**

1. How have you been reaching youth online since the start of the pandemic?
2. In what way did the various social media platforms seem to be effective?
3. Tell me about your experience in adapting or creating sexual health education programs for Native Youth using online platforms. Tell me from start to finish how you went about it.
4. What are the main benefits and barriers for switching programs to an online version in Native communities?
5. What advice would you give to others who are in the position of setting out to advocate for online sexual health programs in their community? What are the top 3 things that could help them be successful?

Quotations for Themes and their Relevant Sub-Themes

| **Themes** | **Sub-themes** | **Principle Verbatims** |
| --- | --- | --- |
| **Competing Community Priorities during COVID-19** | **Food Security & Water Sanitation Measures** | **"I mean, the food insecurity, I feel like we never hear about that on the news. But, um, the food insecurity is a major, major issue, like meeting the basic needs of households has been a primary thing that I've been hearing from, from Native communities and, and others, you know, that they're, they're either doing contact tracing, or they're doing food relief" (Int1)** |
|  |  | **"So, with these care packages that they created was just a combination of like, different like supplies. But I think in terms of security, one of the big challenges was food formula for like, babies and infants and such, toddlers, probably. I know, that was a really big challenge. And it was a big challenge before in communities because of I think just trying to normalize breastfeeding for a really long time. And then when COVID hit, I think a lot of people now sort of under realize, like there wasn't enough formula to get there wasn't enough food, there was not enough wipes. So, then they're like, oh, let's try this breastfeeding thing. And low and behold, you know, they did find that it was more of a natural way and helpful" (Int2)** |
|  |  | **"So I think too, we did learn from another site as a team that when folks were engaging other youth and other activities, they gave out like grubhub gift cards versus doing like Amazon gift cards, or online gift cards. So, I think that may have helped a little bit better" (Int2)** |
|  |  | **"But our center really quickly pivoted to having like to addressing food insecurity as well as COVID related contact tracing COVID testing sites as well. So we give out wellness boxes and both of the communities that are that have RCL, where families that don't have enough food or don't have adequate access to food can get these wellness boxes that are that have a lot of food" (Int3)** |
|  |  | **"We've also built water sanitation stations because a lot of the communities in our that we work with don't always have running water. So, this to be safe during COVID. It's super important right to wash your hands and if you don't have access to running water or you only have some if you have a set amount of water, right? And you've designated that for showering and washing dishes, you may not think that washing hands that Is your priority if you only have a certain amount of water" (Int3)** |
|  |  | **"And we've also been building hand washing stations. So like I was saying earlier, people don't have running water. And so you can ask people to wash their hands, if they don't have running water, they need to save that water to drink and to clean themselves and to, you know, cook their food. So we've been creating hand washing stations that we've also been delivering to our various partners" (Int4)** |
|  |  | **"In some of the communities, you have homes where people don't have access to running water, they don't have electricity, you have a lot of families living together in close quarters, which make things like hand washing and social distancing, and isolation and quarantine almost impossible" (Int4)** |
|  |  | **"We've been doing a lot of food delivery. So we've been getting a lot of donations from different organizations and soliciting donations. And then, you know, these communities where we work are huge, and it takes hours and hours to get to some of the neighborhoods, you know, in some of the cities, so we'll, so our team does a lot of driving to deliver food boxes" (Int4)** |
|  | **Financial Hardship** | **"A lot of young people here are having to take jobs as part of like, meaning to contribute when their parents have lost their jobs, which throws their ability to participate in programs and even their education into peril" (Int1)** |
|  |  | **"And so some of the challenges that were already existing in their communities as far as like, economic challenges, lack of access to resources and some of the rural areas those young people have whose families have been impacted by losing jobs or things like that" (Int1)** |
|  |  | **"They have to deal with food insecurity, and, like, unemployment, so the kids have to help their parents, that is a national dilemma, right, you know, everybody sort of, kind of everything sort of falling away. But the basics, you know, and, you know, it's an unfortunate thing about this pandemic, is it's really just shedding a light on long standing inequities, and deep seated inequities between groups in this country. And you know, it's really just shining light on that. And hopefully, some good will come of it" (Int4)** |
|  |  | **"And a lot of the students some of them might be home, like back in their hometowns. So a few of them, I think they're still in the town of Tucson, but a lot of them probably have stayed home with their families. So but they have other responsibilities with their families, too" (Int5)** |
|  |  | **"And then that coupled with tribes being on furlough, people losing their jobs. Yeah, people, yeah, communities losing their, their youth center, due to having to re-allocate resources to other projects. So it's, yeah, it's been a pretty big impact" (Int6)** |
|  | **Mental Health Impact** | **"And most of our tribes are struggling to support the mental health and well-being of the people they serve. Um, we're already seeing that on both, even here in the Southwest, we're seeing it so that that lack of stability is really hard for young people the like, not knowing what to expect, is very difficult for them to cope with" (Int1)** |
|  |  | **"And I think our we're at real risk of like a widespread mental health crisis for young people, for teens, particularly. So we cannot, we cannot not address that, like young people's lives are at stake when it when it comes to a time like this. So. So that would be like mental health piece is one of my number one thing to do would be to, like, build competence in, in how to do this and be really, really thoughtful about how you plan and prepare and deliver your programming" (Int1)** |
|  |  | **"And so I got to see firsthand and visit with some folks and their rural location and with the youth that I spoke to, you know, I think, like the rest of like, all of us a lot of stress, a lot of anxiety, a lot of worry, a lot of unknown. You know, it's… mental health is definitely an issue" (Int2)** |
|  |  | **"So that, on a community level is devastating. We've lost a lot of elders who are just carrying a lot of wisdom and knowledge about their culture and their beliefs. And in that sense, from a from a cultural point of view, it's been devastating. From a human loss point of view, it's been devastating" (Int3)** |
|  |  | **"And it's a process and things are changing all the time, you know, the, the pandemic seemed to be under control to some degree, and now it's ticking back up. And that changes the landscape, you know, it's going to change, people's willingness to engage online, I'm going to change their thoughts and hopes about the next few months. So it's going to change their mental health and all those things. So it's all I would say, I would say, I don't think I know all the answers" (Int4)** |
|  |  | **"But I think the youth really need us to be there. They all say where they're fine. They're doing okay. But I think the impact of this pandemic can is really on the mental health. A lot of people are not fine. And this is really a big change for the students" (Int5)** |
|  |  | **"So I think the challenges of moving from the physical space to a virtual space is that disconnection from being around folks physically, I think that's one that I hear often is that, you know, with tribal communities in particular, at least from my experience, we've got a big hug of people. I think the mental health impacts for that are going to be pretty huge in the next decade or so. Yeah, so that I that's, I think that the major piece there" (Int6)** |
|  | **Focusing on COVID-19 Response in Clinics and Centers** | **"So a lot of public health clinics have shifted their focus away from some of the core of public health, which is, you know, contraception, they have shifted away from that, because they're, they're having to do COVID. So yeah, quite a bit in even the non-clinical side, like, even in youth programs, a lot of staff have been reassigned to COVID response and contact tracing. And so yeah, a lot of those services are not as accessible" (Int1)** |
|  |  | **"And then one more thing, too, is I do know, there has been a lot of like hesitancy in terms of going into different clinical systems in terms of sexual health care. So getting those like regular checkups, a lot of folks that are a little hesitant to go into the, to their local tribes or communities just because of like, you know, the hospital, and they don't want to get COVID because, again, in some communities, they only have one health clinic space. And so those, you know, probably too, where maybe some patients COVID positive patients go to, so I think those are some things that I think travel communities, but also learning to navigate" (Int2)** |
|  |  | **"Some of these clinics were had staging areas outside of their clinics in order to be able to do intake, whether it was a potential COVID patient, or what have you. And in during the wildfires, the air quality was so severely unhealthy, they just shut everything down. So, um, access to services is still an issue, but you know, trying to communicate to youth that they have this option, and the conduit for those folks are facilitators are there" (Int2)** |
|  |  | **"And through our partnerships with IHS, we've also been asked to help them with other COVID related efforts. So, some of that, but that all that work is new, and not necessarily what our team was doing prior to COVID. And it's constantly changing, and in flux that both of both the communities that we work with the most closely, there have been weeks where we have our team back, and it's almost as if you know, it's not COVID, staff attendance and availability, but then, you know, the surge and we lose some of our staff members to COVID related for necessary COVID related work" (Int3)** |
|  |  | **"But our center really quickly pivoted to having like to addressing COVID related contact tracing COVID testing sites as well. So we have like a couple of testing sites like regularly that happened in the community where we, our staff are the ones that help, IHS, or we ourselves, set them up to do testing in the community, we have what we call them, and then we help with contact tracing as well" (Int3)** |
|  |  | **"Yeah, I mean, our center really pivoted to be like public health responders, you know, we we, again, knowing that what we know about the communities where we work and all of our colleagues that we've had for, you know, 40 years, we knew what how this was going to impact them. So we really, you know, we have a lot of infectious disease research experts within our center and also within the school. And so we look to them to really create like an on the ground plan. So a lot of our staff, you know, were trained in contact tracing. And in COVID, testing, they've been doing that they've been part of contact tracing teams, they've been part of, you know, swabbing and testing teams and mobile clinics" (Int4)** |
|  |  | **"I think they're slowly going back to opening up their health centers, but I think scheduling a lot of is virtual zoom schedules. The WIC department is, I think still not seeing their patients and their families face to face yet so I'm not sure how it's going to be looking by the beginning of next year" (Int5)** |
|  |  | **"So it's telehealth. And, yeah, so there's been recommendations for youth to you know, get in touch with their telehealth. And then we don't know how it's going, how that's actually translating, and how that's actually working if it's being successful or not. So yeah, there's been content that we're creating around how to access sexual health resources. But yeah, from tribe to tribe, it's very different" (Int6)** |
|  | **Sexual health as a Secondary Concern** | **"So a lot of our program has had to shift from that hyena thinking about like the Maslow's hierarchy of needs, right, we're shifting away from the sex ed aspect all together, and we're doing, you know, our teams are doing food deliveries and supply deliveries, PPE stuff" (Int1)** |
|  |  | **"And now we're seeing a federal response, saying here is a curriculum, a list that can be utilized for some of these programs. I think what's interesting is that it's just not the silo of physical sexual health. It's more holistic, mental health, social health, cultural health and physical health" (Int2)** |
|  |  | **"But reproductive health curriculum is considered extra, and therefore, sometimes gets cut not all schools, but excuse me, in some schools. As far as that that's just been challenging" (Int3)** |
|  |  | **"But we had some interested people who we kind of kept like a list of. And so those participants actually were the ones that were interested in RCL. And they kind of felt that despite the pandemic, they still wanted to take part. And that kind of was our motivation to say, okay, good, the community is still interested in this, they still want this program, despite everything that's going on. Yeah, that was, I think, not surprising, because we know that we didn't think people wouldn’t be interested. But we just knew that people's priorities may have shifted" (Int3)** |
|  |  | **"And maybe people are so worried about other things that sexual health is probably not a priority for them" (Int5)** |
|  |  | **"It's definitely been placed on the back burner" (Int6)** |
|  |  | **"You know, working at the tribal center, its COVID response, and so it was prioritizing needs, and the needs weren't sexual health, per se, it was making sure that everyone was safe that their families receive. So it was refocusing the sexual health content to be mostly around mental health focus, and relationship building. So adapting whatever topical area might have been included in a sexual health curriculum, say healthy relationships, and reframing that message to be in a more informal space that allowed to build relationships. That was a huge impact" (Int6)** |
| **Moving to Web-based Programming: Skills, Training, Support** | **Adaptation of Programs to the Online Platform** | **"And so, writing a grant from the perspective of like, we don't, you know, it'd be new, we didn't know how to plan and how to write, you know, for that. So, we just wrote it as if we were implementing as normal and figured we would figure out the adaptation process later. And so, so that's been kind of a challenge now that we've been awarded, we're trying to figure out how to how to implement virtually how to coordinate with a school who's doing blended learning and meet their needs, as well" (Int1)** |
|  |  | **"It can be very quick to open up a zoom account and get started on zoom. Right? You can do it in minutes. But that doesn't mean that you're prepared to implement a program online. And so. So, I think that one of the challenges is that people are going to, I think, initially hastily put their programs together, and they're not going to be good. And they're not going to actually meet the needs of young people" (Int1)** |
|  |  | **"And so, you know, very quickly, we had to start adopting our own programming for virtual settings. And, you know, kind of identify which components of our work we're going to get continue happening in community settings, because most of our tribal clinics closed, our tribal schools closed, public schools closed. So, kind of being in touch with our tribal health educators and our clinic staff to see which services and programs were continuing, what was being paused, what priorities they had for moving forward and trying to respond to those" (Int2)** |
|  |  | **"So, we cancelled that camp and quickly we had to figure out how to do RCL. So in in light of COVID, we decided that it may make sense to try a pilot program of RCL being conducted virtually. We have not done ourselves in a virtual format before, we've been used, working with RCL for quite a while now several years, if not more. But it's never been done virtually. So, this was brand new for us. And it was… it's a program that requires a lot of in-person activities" (Int3)** |
|  |  | **"For the current way that we implement the respecting the Circle of Life program, it is with federal funds, but it's, there's more flexibility with this particular federal funder, like there, they were really encouraging of us to try a virtual implementation, which we've been doing for the last few months, we've been trying to deliver the program via zoom. And that's been, you know, a real learning process. But, um, you know, really, most of our programs are pretty much halted with the exception of respecting the circle of life, which you've been trying to do virtually" (Int4)** |
|  |  | **"Yeah, I think it was just a huge delay in trying to figure out what to do in terms of adapting content. So some of the content that we work with pre-COVID was in an online platform. And so obviously, that translated easier. But even then, they most folks were still meeting with youth in some capacity physically, even though it might have been, you know, multimedia. Yeah. Technology that they were using. So yeah, lots of adaptations" (Int6)** |
|  | **Lack of Sufficient Time and Staff Support in Stressful Adaptation Process** | **"And they're, like requesting it, they're begging for support. And it's all over the country. I mean, the level of support that people need right now is substantial, I'm working like seven days a week, for weeks on end, like during, during this crisis, because the level of support that communities need in order to adapt and to try to function at all, in supporting young people's needs is substantial" (Int1)** |
|  |  | **"And so yeah, that has been an enormous challenge, because it's not like we can say, Oh, just shift everything to these online tools. We first have to teach the tools and how to use them and get some basic skills under their belt and then start to look at, you know, the other piece that comes after that is that I'm a curriculum specialist" (Int1)** |
|  |  | **"So, although some of these were presented in person, there was a lot of adaptation to go online, and what that look like, so a lot of trial and error, a lot of challenges and a lot of different avenues of communication and having to be also creative, and also still innovative too (Int2)** |
|  |  | **"Secondly, was how to coordinate our schedules and utilize a virtual platform such as zoom, and become zoom savvy for us as professionals, and I can tell you, I think we've talked about it, we've been more busy now in a virtual setting than we have when we were in the office" (Int2)** |
|  |  | **"We also had time constraints because we wanted to get this up and running and stay fairly focused, and to do everything all at once, it just wouldn't be feasible and also it may not create kind of a cohesiveness" (Int3)** |
|  |  | **"I know a lot of tribal employees were told to work from home, which is a lot of them don't have the internet to work from home. So I think a lot of that was trying to adapt to it and try to find resources that can assist them" (Int5)** |
|  |  | **"So, we're sort of like, we keep telling people, we're like pulling the curtain back a little bit to show you the background of what this looks like to run a session like this, because it helps them build those skills and understand like, oh, here's how you, you know, use a quiz or a poll or something on zoom or another online platform. And yeah, just introducing some of those basic tools. So, we've been doing that. And then, we're also writing a guide that we've been working on for like six weeks. But it's, it's tough, because we want it to be robust enough to actually support communities in that adaptation process" (Int1)** |
|  |  | **"Trainings are great. We've been doing a lot of trainings and a lot of recorded sessions on YouTube. That's been a big, I think, a facilitator for us in our programming" (Int2)** |
|  |  | **"The second thing about this, though, is a technology education curve, how including myself in that group, you know, learning how to how to zoom, you know, zoom one-on-one, and then working with youth who may not know zoom, they only know FaceTime, you know, or Facebook Live" (Int2)** |
|  |  | **"So, we had to incorporate an entire tech training on how to use zoom and how to use like the internet to do a lot of things. And I mentioned our team, our paraprofessionals, which means they're from the community, but they may not always have all of the skills. So, for us training is really important to make sure that we're helping our team members kind of reach their best potential, so they can do all of these things" (Int3)** |
|  |  | **"And so we have a really strong curriculum team that does all the trainings with the, with the facilitators, and then we have a very robust system for ensuring that they feel ready to go and so that just includes a ton of role playing with each other with us. There's an exam that they have to take at the end. There's also like a competency checklist that they take at the end just to ensure that they understand the content and feel prepared for the delivery, but they also understand, you know, how to be proficient with zoom and to troubleshoot different tech challenges that will come up along the way. You know, so I think that I think that we actually are doing well" (Int4)** |
|  |  | **"I think it was, say it was both kind of learning how to get in information and providing it in a virtual setting, a lot of it was changes because all of our, our work, we do a lot of face to face training" (Int5)** |
|  |  | **"So holding trainings for the adults to try out breakout rooms, and try the whiteboard and the annotation features and trying out other apps, you know, that make online learning a bit more engaging" (Int6)** |
|  | **Youth Missing One-on-One Connection** | **"But I think even if we are able to convert our programs to being fully virtual, I think there is still going to have to be some in person components to really like, engage people and get them excited about the program" (Int3)** |
|  |  | **"So we have had, we have had some zoom sessions, where there are, you know, siblings together with their parent, and there's a couple kids, but their original program, you know, kids are in groups, and they're doing all these fun activities, and they're laughing, and they're really engaged. It's very much like an interactive process. And so, you know, some of the activities that were like that originally that we knew we couldn't do a resume we either took out or kind of modified" (Int4)** |
|  |  | **"Yeah, so that was the I think one of the impacts was a lot of the students were required to meet, like, they had like a little house on campus. So that was kind of like, their place to hang out together. And it was like a kitchen for them. So sometimes they would have food there. And especially when you're a college student, you know, sometimes you don't have the money. Yeah, but since that pandemic happened, a lot of it is virtual. So some of the students are actually probably missing that one on one connection" (Int5)** |
|  |  | **"But for tribal communities, the in-person part is such a major piece. And I don't see that changing in any capacity, I do think that the virtual piece will be really amped up, and I think it will be smoothed over quite a bit" (Int6)** |
| **Recruiting Youth** | **Using Social Media Platforms for Youth Outreach** | **"We've been using Instagram quite a bit. It's been I've been kind of waiting for Instagram to go out of fashion with young people. And so far, it has not. I've been surprised. We tried on a project I'm working on, we tried to get young people to do some things on Tick tock, and they were like, oh, we'd rather do it on Instagram" (Int1)** |
|  |  | **"So, I think that they, like previously, young people wouldn't be caught dead on Facebook. And now they are going on to Facebook a little more to get information about things like particularly their programs and stuff" (Int1)** |
|  |  | **"So, we run kind of a multimedia Health Resource for Native youth that includes the website, Facebook, Instagram, Twitter, and then you know, so we can kind of see the analytics on those channels, and what sort of messages are getting the best reach and impressions" (Int2)** |
|  |  | **"Our facilitators themselves have used their personal social media to reach out to youth and families that they know in their communities, so they're able to post things on their Facebook page, or on like, like, there's yard sale pages and things like that, that are more community focused. So, they are able to post there. Facebook has been really helpful. I think that's the main tool we've used. They also I think, had a little bit of success with Instagram, we're trying to see if we can get to tick tock started" (Int3)** |
|  |  | **"We use Facebook and Instagram; we've we haven't quite gotten into tik tok yet" (Int4)** |
|  |  | **"We just have Facebook right now" (Int5)** |
|  |  | **"Yeah, um, so all of the social media channels, mostly focused on Instagram, YouTube, as the primary focus, and then Facebook, mostly for getting content to the older, the adults" (Int6)** |
|  |  | **"But generally, every year or so we'll do a tech survey, just to make sure the platforms that we're using are current and the content that we're posting is relevant. And yeah, so that's why we're focusing on Instagram in particular" (Int6)** |
|  | **Differences in Online Program Youth Participation Rate** | **"Yeah, there’s definitely… the recruitment and the retention in a virtual setting has is… versus an in-person implementation, yes, there is a dramatic difference. Only because of the fact that you know, that we're trying to ourselves as an organization, and educators trying to find resources and tools that they can implement, if they were to implement a curricula, trying to adapt that. The upside that we're seeing is an uptick in our social messaging for youth. That's a very popular resource" (Int2)** |
|  |  | **"We did see a little slight decline since the virtual setting for schools that some of the kids didn't come back. So we don't we don't know where they went" (Int5)** |
|  |  | **"Yeah. And the tribe in particular that I'm thinking about that we are working pretty closely with the sexual health curriculum, their participation had dropped off pretty dramatically, and most of the youth that they were seeing was only on a mental health counseling basis" (Int6)** |
|  |  | **"But we, you know, yeah, part of it is, I think the programs that are going to be successful at recruitment is programs that are operating through a school, it's much harder already, like some of my teams in Michigan have been trying to get the young people together, and they're just now getting to the point. And it's because school has created this, like, sort of organizing opportunity, like kids are having a more structured day than what they have over the summer. And in the early days of the shutdown" (Int1)** |
|  |  | **"And, you know, kind of identify which components of our work we're going to get continue happening in community settings, because most of our tribal clinics closed, our tribal schools closed, public schools closed. So, kind of being in touch with our tribal health educators and our clinic staff to see which services and programs were continuing, what was being paused, what priorities they had for moving forward and trying to respond to those" (Int2)** |
|  |  | **"And our team kind of talked to schools, they talked to a superintendent, they were trying to figure out how schools are going to be doing things even though it was the summer, their summer schools. So, we wanted to see what kind of how that would make sense in the community. Another thing to consider is that site is very rural, and very remote and very impoverished. And if we did this program virtually, would that be a barrier, right, having access is crucial and important, especially in a virtual program. So that was one of the things that we had asked our staff to kind of look into a bit more to see, you know, there's different ways that we can make sure you get internet access, is internet going to be a problem that comes up recruiting" (Int3)** |
|  |  | **"Yeah, I think, I think with schools closing, you know, for programs that are offered in schools, you know, that made it impossible" (Int4)** |
|  |  | **"Some of them were talking about the impact of schools, not being able to go in schools to educate. And then that barrier, and also kind of a lot of the sexual health education is one on one, I guess. And I don't know that people are more open to be talking one on one to somebody about sexual health. But more the outreach has slowed down. Now they have to find a different method of outreaching" (Int5)** |
|  | **Distribution of Flyers in Community Locations** | **"That's one of the effective ways. I mean, flyers in a place that people are still going so um, you know, in in some of the smaller Native communities like that, there may be like, people actually go and visit a bulletin board or they go and visit like something in a primary building. Or especially like, where things like, you know, the food pickups are happening, a flyer on a bulletin board or a flyer in a location like that can be, you know, a good tool for getting the word out" (Int1)** |
|  |  | **"I think there is still going to have to be some in person components to really like, engage people and get them excited about the program. Because I think also being in research there's you have to when you advertise things, right, it's a lot of information you have to make sure you include, which is all good, but it's not necessarily the most exciting like recruitment flyers" (Int3)** |
|  |  | **"We also do continue to distribute, you know, flyers and community locations, we put flyers on people's cars. We have some billboards, like electronic billboards, where we've bought out time for, you know, recruitment materials. So, you know, we're really just trying to work the channels that are existing in the community" (Int4)** |
|  |  | **"So and especially working with tribes, a lot of tribes like to travel out because a lot of them don't have the internet resources. So that was another impact was trying to find the communication source because a lot of it was is going to be email, but kind of creating flyers and resource lists was the other…was another thing" (Int5)** |
| **Challenges for Implementation in Household Environment** | **Challenge of Youth Program Participation from Home Environment** | **"The parents will say, oh, I'm going to put or sign up all four of my kids who are all in that age group to be in this class together, and they're going to all join. So, we weren't sure how that would work out. We weren't sure. Would youth just sign in from different devices? Because that makes polling and these interactive features really doable? Or will you if they're in the same house, if they're siblings, for example, or cousins, or relatives or you know, a germ unit that are all seeing each other regularly? Are they going to just sign in from one computer? Because then that throws some of the activities we planned, it changes them a little bit? Right?" (Int3)** |
|  |  | **"And we've all kind of heard this, like zoom fatigue is such a thing where you're just on video calls all day, and you're exhausted. So, we didn't want this to be one more thing that a kid has to do that it's something that they want to do" (Int3)** |
|  |  | **"And part of what is, what's nice about the camp model is the kids can kind of work through those nerves together. And there's like some fun games they play and things they can do to kind of practice things, some of the phrases and the words that are a little bit uncomfortable. And we do some of that in the virtual model. But it is it is more challenging when it's like a one on one situation versus like a fun group at a camp" (Int4)** |
|  |  | **"So but they have other responsibilities with their families, too. So right now I am mentoring one person. And my communication with her is very difficult, because she lives far in New Mexico so and she kind of has family that she has to watch. Since she's not in school down in Tucson. She's doing her virtual schooling from there. So that is a little challenge" (Int5)** |
|  |  | **"But you know, there's lots of need around, you know, with intergenerational families sharing technology. So you know, maybe the youth was assigned a computer that or you know, a tablet that did have data on it, but then that youth might have to be sharing it with siblings or the adults. And then also family schedules. So you know, if there's a family and this youth is supposed to be on a sexual health lesson, but then mom needs to go grocery shopping, just take all the kids with her" (Int6)** |
|  |  | **"So, for instance, young people that are living in homes that aren't safe, and that may be like participating in a sexual health program, in a household where that is not accepted. Whereas a young person could have previously like said, Oh, my after-school program is like, spirit club or something, when it was actually a sexual health program. Um, you know, now, if they're doing something at home, they are in a home where other people may see what they're doing and know what they're up to. And in homes that are not safe to do that, that can be a real barrier" (Int1)** |
|  |  | **"Even on clinical services, we're seeing that like, you know, pediatricians and doctors that are working in adolescent health, they're saying, like, I can't talk to, you know, these young people in a virtual visit in a safe way. Or we're having to ask them, like, is this is this a safe time to talk? You know, do you have privacy right now? Which is, you know, it's hard. We have previously, you would have asked the parents to just leave the leave the exam room, but yeah, so. So yeah, I think that it sort of like, it really depends on young people situation at home, and it's a lot more, there's a lot more room for young people to be in trouble for participating in the sexual health program. whereas previously, their parents wouldn't be there watching what's happening in the program. And parents typically are not as open minded about these issues, especially in Texas, and in you know, rural Michigan, conservative Michigan. These are not communities where it's easy to do sexual health and so, so yeah, I think that can be a real barrier as well" (Int1)** |
|  |  | **"I think, like I mentioned earlier, and I jokingly said it before, it kind of ropes back to this, is the adults learn right along with the youth in a more modern context, and neutralizing any fear. I think it's just the outside noise that makes it seem like it should be taboo. And it's really not, it's just, that's why with this generation through the technology and the conversation, they're taking it and owning it. Just the older folks, that's gotta let them learn and, and let them live. And we offer that guidance, but they make the choices" (Int2)** |
|  |  | **"And that last session is to hopefully increase communication between the youth and the parent. We also do a condom demonstration and talk about safe sex and all of these things with the parent and youth together" (Int3)** |
|  |  | **"As much as we love tech, on the other hand of it is like people connection, people interaction is so important, especially for creating safe spaces for Reproductive Health knowledge. And like creating this relationship between the user and the facilitator, the youth and other youth like youth are missing out on learning in larger classroom settings" (Int3)** |
|  |  | **"And the kind of like, the taboo of tribal communities, of talking about sexual health, and the embarrassment of condom use, and even taking one or knowing somebody is passing out that information" (Int5)** |
|  |  | **"Now, a lot of rural youth also have the challenge of that they don't have internet connection, stable internet connection, or high-speed internet, which is really shocking that it's 2020" (Int1)** |
|  |  | **"But we've also heard collectively, what, what the needs are. And, and that's pretty compelling from the field, infrastructure, weeds. You know, there is a digital divide out there. So not everybody can utilize a computer and a laptop or enroll America in most places. So that's been pretty compelling" (Int2)** |
|  |  | **"So that all to say that not all youth would have computers at home, not all youth would have internet access at home. And if we did this program virtually, would that be a barrier, right, having access is crucial and important, especially in a virtual program. And so, we definitely wanted to be mindful of that. So that was one of the things that we had asked our staff to kind of look into a bit more to see, you know, there's different ways that we can make sure you get internet access, is internet going to be a problem that comes up recruiting" (Int3)** |
|  |  | **"You have to make your program virtual, because how else would you reach these people? I mean, the challenge of it is, technology is not perfect. So, having like losing internet is something that you have to take into account, and you miss the human component, right" (Int3)** |
|  |  | **"So and especially working with tribes, a lot of tribes like to travel out because a lot of them don't have the internet resources" (Int5)** |
|  |  | **"Right now is the internet. That's the big thing. Some of them were provided internet services. Some of them didn't have a computer or a laptop" (Int5)** |
|  |  | **"But you know, there's lots of need around, you know, with intergenerational families sharing technology. So you know, maybe the youth was assigned a computer that or you know, a tablet that did have data on it, but then that youth might have to be sharing it with siblings or the adults" (Int6)** |
|  | **Low Bandwidth and Network Connectivity Issues** | **"But also, we wanted to be mindful of the bandwidth that people have like literal Internet bandwidth. Because we knew that like having lots of different websites to click out to might not work, it's probably really confusing and some of our youth as young as 10" (Int3)** |
|  |  | **"But your mileage may vary some flat lessons, you know, and write in an hour, 45 minutes, or 45 minutes or an hour mark and some and take an hour and a half to take, you know, to happen, which has sometimes to do with like network connectivity issues, because our team sometimes will spend a good amount of time like their internet will cut out or the youth’s internet cut out. And so, then you spend some time with logging back in or like making sure things are working" (Int3)** |
|  |  | **"But it's challenging, you know, as you pointed out, like we have places where the internet is really unreliable, and it's really spotty. And so, I think that's really challenging when you know, the session start and then maybe they drop, and they've got to pick them back up again. And, you know, if that happens too many times, you're that that youth is gonna, they're gonna sign up" (Int4)** |
|  |  | **"Some tribes don't have the broadband that connects certain phone services. For internet, so I know some communities have posted up like hotspot locations for people to use" (Int5)** |
|  |  | **"Yeah, yeah. So it's been a lot of technical support for tribes that have varying capacity and resources. So it really varies from you know, rural like not being able to find hotspots, no IT, multi-generational families within a household to urban, in some of the urban areas like Portland" (Int6)** |
|  |  | **"Yeah, I think overall, we know that youth are able to access and have yet are able, yeah, have technology, resources. And so if that's the case, then this is the best platform to do it. So yeah, and I think it's, you know, it's a pretty small number of folks who have absolutely no IT capacity whatsoever, it seems like most tribes, at least, you know, can lend a laptop and a thumb drive. And it's not a perfect scenario. But that's yeah, an adaptation that folks are making" (Int6)** |
|  |  | **"So there are COVID relief funds, and some of those funds are going out to tribes to, in most tribes that sounds like are able to purchase IT equipment, and either give it to families or either lend it to them, they have to sign out for it" (Int6)** |
|  | **Dealing with Youth Internet Access** | **"One of the things that I've been talking to a few folks about is that, you know, what is the commonality in most programs that I've delivered over again, the last, like 15 years of my career has been that transportation is consistently an issue everywhere you go. So, even if you operate an after-school program, young people, they don't have a ride home can't stay, they've got to catch the bus to get home. Young people in rural communities are often not able to participate in programs because they can't get a ride to wherever the program is happening" (Int1)** |
|  |  | **"And then at least in schools, they can still access the program and complete everything, and they don't have to worry about internet connection or like the bandwidth" (Int4)** |
|  |  | **"Some of them didn't have a computer or a laptop. So they they're currently some of them are renting a laptop from the school just to get through" (Int5)** |

| Codebook | | | | | |
| --- | --- | --- | --- | --- | --- |
| **Code Family** | **Code Family Description** | **Code Name** | **Code Description** | **When to Use/Subject/Notes** | **Part of the Interview** |
| **Programs** | **Pertain to Native adolescent sexual health programs** | **Prog-AdolescentSexualHealthEducation** | **This code describes the diverse adolescent sexual health education programs available for Native communities** | **1. Type of adolescent sexual health program 2. Sexual health programs delivered for Native youth 3. Activities: In-person, online, hybrid** | **Question #1 Programs: *Which adolescent sexual health education program(s) have you used in your community? What was your primary role?*** |
|  |  | **Prog-PrimaryRole** | **This code describes the primary role(s) carried out by experts in Native adolescent sexual health programs** | **1. Expert introductory section 2. Primary role carried out in program(s) 3. Mention if multiple roles are carried out** |  |
| **EXPCOVID-19-PERSONAL** | **Pertain to the overall experience of experts in dealing with COVID-19 in their field of work** | **EXPCOVID-19-Personal-Remotework** | **This code describes the personal experience of experts with COVID-19 when switching to working remotely** | **1. Working remotely instead of in-person 2. Distinguish between experts who have worked virtually prior to COVID-19 and those who are new to the virtual platform** | **Question #2 Experience with COVID-19: *Can you please start by describing your overall experience with COVID-19 in your community, particularly in your field of work?*** |
|  |  | **EXPCOVID-19-Personal-VirtualSet** | **This code describes the adaptation process to the virtual setting during COVID-19** | **1. Adapting tools, lessons, and interactive sessions 2. Software needed 3. Fast-paced adaptation 4. Focus on short-time frame of virtual adaptation** |  |
|  |  | **EXPCOVID-19-Personal-TribalComm** | **This code describes the ongoing communication process with Native tribes during the COVID-19 pandemic** | **1. Communication with tribal health educators and staff** |  |
|  |  | **EXPCOVID-19-Personal-AssRead** | **This code describes the assessment of the readiness of Native communities to be involved in different types of programs** | **1. Assessment of readiness 2. Assessment of needs** |  |
|  |  | **EXPCOVID-19-Personal-ShiftRole** | **This code describes the shifting roles of project staff to the COVID-19 taskforce** | **1. Involvement in contact tracing 2. Focus on COVID-related work** |  |
|  |  | **EXPCOVID-19-Personal-ZoomSavvy** | **This code describes the importance of becoming zoom savvy in a short timeframe to keep up with the pandemic** | **1. Becoming zoom savvy 2. Challenges of learning a new platform 3. Some experts did not have the chance to use zoom before the pandemic hit since most Native communities relied on the in-person component** |  |
|  |  | **EXPCOVID-19-Personal-SchoolCoord** | **This code describes the process of coordinating with schools to switch to the online platform** | **1. Adaptation process from in-person to online and modes of delivery with school staff 2. Most programs are delivered through schools for wider recruitment purposes. Since schools have switched to the online platform, Native adolescent sexual health programs are forced to follow the same format** |  |
| **EXPCOVID-19-Community** | **Pertain to the overall experience of Native communities in dealing with COVID-19** | **EXPCOVID-19-Community-Experience** | **This code is used for all inquiries related to the impact of COVID-19 on Native communities in general, and on the delivery of sexual health programs in these communities in particular** | **1. Socioeconomical and health challenges 2. Problems caused by COVID-19 in the community of interest** |  |
|  |  | **EXPCOVID-19-Community-InternetAccess** | **This code describes the impact of COVID-19 on the smooth delivery of programs due to limited internet access among Native communities** | **1. Accessbility to the internet 2. Residing in remote areas** |  |
|  |  | **EXPCOVID-19-Community-ComputerAccess** | **This code describes the limited access to computers by Native youth** | **1. Inability to afford computers at home 2. Reliance on computers at school** |  |
|  |  | **EXPCOVID-19-Community-EconChal** | **This code describes the economic challenges imposed by COVID-19 on Native communities** | **1. The necessity of youth to take jobs to help out their families 2. Loss of jobs among parents in rural areas** |  |
|  |  | **EXPCOVID-19-Community-TribeStruggle** | **This code describes the continuous struggling of tribes and the devastating impact of the pandemic** | **1. Community hit-hard 2. Devastating at cultural and community level 3. Loss of elders** |  |
|  |  | **EXPCOVID-19-Community-ShiftofProg** | **This code describes the shift of programs from a sexual health aspect to meeting the basic needs** | **1. Shifting from sexed to food delivery, supply delivery, and PPE** |  |
|  |  | **EXPCOVID-19-Community-MentalHealth** | **This code describes the impact of COVID-19 on the mental health of Native communities** | **1. Emotional toll on youth 2. Mental health issues in tribes 3. Struggle of youth to balance education while helping parents** |  |
|  |  | **EXPCOVID-19-Community-HesitCare** | **This code describes the hesitancy of Natives to seek care from clinics during COVID-19** | **1. Fear of getting COVID-19 2. Hesitancy to seek reproductive care** |  |
|  |  | **EXPCOVID-19-Community-PeakSTDs** | **This code describes the expected peak in STDs caused by COVID-19 due to the lack of focus on sexual health of youth** | **1. Perception on sexual health guided by misinformation 2. Parents are not aware that their kids are sexually active 3. Lack of data to describe potential peak 4. Sexual health not considered as a priority since start of pandemic** |  |
| **Consequences** | **Pertain to the main consequences caused by COVID-19 on ongoing sexual health education programs in Native communities** | **Consequences-VirtualAdapt** | **This code describes the consequences of COVID-19 regarding the fast-paced adaptation of in-person programs to the online setting** | **1. Going online by trial and error 2. First time trying the online platform 3. Changing entire format 4. Difficulty managing technology 5. Making sure that program is culturally appropriate is challenging within short timeframe** | **Question #3: *What were the main consequences caused by COVID-19 on ongoing sexual health education programs in Native communities?*** |
|  |  | **Consequences-AdaptandPilot** | **This code describes the adaptation and piloting of previously delivered in-person activities to the online platform** | **1. Adaptation of physical games to the online setting 2. Building skills in relation to creating polls, quizzes 3. Community of practice and development of guidelines to improve staff skills 4. Quick adaptation of in-person tools was needed to maintain integrity of program** |  |
|  |  | **Consequences-StaffSupport** | **This code describes the need to continuously support staff members during the stressful process of virtual adaptation** | **1. Level of support needed by staff 2. More busy in a virtual setting than in the office 3. Staff stressed and under a lot of pressure to adapt an entire in-person program to an online platform 4. Under stress to learn new tools and manage technological challenges** |  |
|  |  | **Consequences-CultTailored** | **This code describes the need to culturally tailor programs and activities to meet the needs of diverse tribal settings** | **1. Culturally tailored to activities 2. Need to be patient to work with diverse tribal settings** |  |
|  |  | **Consequences-FlexStandards** | **This code describes the need to meet only the basic requirements rather than aim high to reach all standards when switching to the online platform** | **1. Letting go of high standards 2. Meeting basic level standards and supporting people instead** |  |
|  |  | **Consequences-FlexDeadlines** | **This code describes the flexibility of deadlines and timelines generated by the impact of COVID-19 on communities in general** | **1. Sympathy of federal government and funding agencies 2. Extension of deadlines and timelines** |  |
|  |  | **Consequences-CommInvolvement** | **This code describes the increased involvement of communities in the development and adaptation of online programs as a result of COVID-19** | **1. Involving the community in surveys to get a sense of interest 2. Getting ideas from community members on how to proceed 3. Interest to participate despite switch to the online platform 4. Involving community members in adaptation of programs to learn what needs to be done to keep them engaged** |  |
| **Challenges & Barriers** | **Pertain to the main challenges and barriers affecting the ability of sexual health program delivery to youth** | **ChalBar-SexEdPrgm-Unknown** | **This code describes the fear of sexual health education experts from the unknown related to managing programs and switching to the online platform during the pandemic** | **1. Not knowing what to expect in terms of severity and number of cases 2. Delivery of program not as planned 3. People working in sexed are not sexual health experts** | **Question #4: *What are the top challenges and barriers affecting your ability to provide sexual health programs to youth in your community?*** |
|  |  | **ChalBar-SexEdPrgm-Recruitment** | **This code describes the recruitment challenges imposed by COVID-19 on ongoing adolescent sexed programs** | **1. Dependence on schools for recruitment opportunities 2. Difficulty in reaching out to youth living in rural areas 3. Dramatic difference between recruitment and retention in a virtual setting versus in-person implementation 4. Uptick in social messaging for youth for recruitment purposes** |  |
|  |  | **ChalBar-SexEdPrgm-Disconnect** | **This code describes the struggle of sexed experts with connecting remotely to carry out the needed tasks associated with the program** | **1. Challenge of being disconnected 2. Access to network from home 3. Making sure that the needed equipment is available when working remotely** |  |
|  |  | **ChalBar-SexEdPrgm-SkillsLacking** | **This code describes the lack of skills among some paraprofessionals to deliver the program to the online platform** | **1. Tech training is needed due to difficulty with zoom management 2. Limited skills of paraprofessionals from the community** |  |
|  |  | **ChalBar-SexEdPrgm-Longsessions** | **This code describes the need to cut down the program to shorter sessions when adapted to the online platform to increase retention** | **1. Sessions were too long when adapted to the virtual platform 2. Ended up making it a longer program to fit the platform 3. Online sessions shortened from two hours to an hour long 4. Lesson duration influenced by connectivity issues** |  |
|  |  | **ChalBar-SexEdPrgm-Taboo** | **This code describes how sex is perceived as a taboo in Native communities and thus creates a challenge for participation** | **1. Talking about sex in Native communities is a taboo 2. Kids would join in on a computer surrounded by family members who can hear everything happening in that session 3. Awkward to learn when parents are around** |  |
| **Access to SexEd** | **Pertain to the impact of COVID-19 on the ability of youth to access sexual health programs** | **AccesstoSexEd-Transportation** | **This code describes the struggle of Native youth to get a ride home after attending sexed programs** | **1. Can't stay for after-school programs 2. Young people in rural communities often can't go to any programs because they don't have a ride home and can't stay 3. Young people might have been disconnected from other programs due to lack of transportation 4. Transportation has been a barrier for decades** | **Question #5: *How has the COVID-19 pandemic impacted youth's ability to access sexual health programs?*** |
|  |  | **AccesstoSexEd-InternetConn** | **This code describes the instability of the internet connection in rural areas where Native youth reside** | **1. No stable or high-speed internet** |  |
|  |  | **AccesstoSexEd-AccesstoZoom** | **This code describes the limited access to zoom among Native teens and their parents** | **1. Limited access to zoom platforms among parenting teens 2. More personnel level using private messages through their private accounts on Facebook 3. Trying to use Facebook and zoom despite challenges** |  |
|  |  | **AccesstoSexEd-CutofSexEdSchool** | **This code describes the cut of sexed programs in schools to focus on major subjects and decrease the burden on both students and staff** | **1. Schools in Arizona are not mandated to provide reproductive health education 2. Schools focusing on testing and other COVID-related things that are measured by certain metrics  3. To reduce burden on youth and stafff, schools have cut reproductive health classes 4. Focusing more on English, math, history, and other credits 5. Reproductive health curriculum is considered extra** |  |
| **Access to Reproductive Healthcare** | **Pertain to the impact of COVID-19 on the ability of youth to access reproductive healthcare services** | **AccesstoRepHealth-ShiftinFocusClinic** | **This code describes the shift in focus in clinics from offering reproductive healthcare services to solely focusing on COVID-related services** | **1. Public health clinics shifted away focus from contraception because of COVID 2. Services were not that accessible before COVID and now they are even less accessible 3. Lack of opportunity to access some core protective and prophylactic things that can support people's sexual health 4. Operations had to close down and become restrictive 5. Clinics had staging areas outside to manage intake of patients during COVID 6. Shut down everything during wildfires because the air quality was so unhealthy 7. Trying to tell youth to reach out to community health aids and behavioral health experts since they act like youth council or peers** | **Question #6: *To what extent have youth been affected in accessing reproductive healthcare services in your community during the pandemic?*** |
|  |  | **AccesstoRepHealth-GapinCondoms** | **This code describes the interruption caused by COVID-19 on the delivery of condoms to Native youth in diverse communities** | **1. Used to offer on the We R Native website condom distribution 2. Sign up for condoms to get them by mail 3. Put a hold on condom distribution because couldn’t go to the office to put those packages together** |  |
|  |  | **AccesstoRepHealth-LimitedAppt** | **This code describes the limited appointments given by clinic for reproductive health services or related checkups due to being overwhelmed with dealing with COVID cases** | **1. People in the community are trying to be very safe and cautious by limiting outside interaction 2. Some reservations have been locked down 3. People are not allowed out 4. Don't know how often people are going to have their regular check ups during COVID 5. Not sure whether people are still keeping reproductive health appointments 6. Don't know whether appointments are being cancelled or postponed due to COVID** |  |
| **Actions to Mitigate COVID-19 challenges** | **Pertain to actions taken to mitigate and alleviate the burden caused by COVID-19 in Native communities** | **ActCOVID-Foodsecurity** | **This code describes the actions taken to address the issues associated with food insecurity** | **1. Meeting the basic needs of the households is a primary thing for Native communities 2. Care packages with different supplies 3. Giving out grubhub gift cards rather than Amazon gift cards to deal with food insecruity 4. Giving out wellness boxes in both communities receiving RCL 5. Families don't have enough food or don't have adequate access to food 6. Having a food sovereignty coalition trying to address and respond to community needs assessments  7. A whole team works on food insecurity at the organizational level** | **Question #7: *What were the actions taken to address the current COVID-19 related challenges?*** |
|  |  | **ActCOVID-Breastfeeding** | **This code describes the switch to breastfeeding to meet the basic nutritious needs of Native infants** | **1. One of the biggest challenges was baby formula 2. Not enough formula or wipes when COVID-19 hit 3. Turning to breastfeeding and breast milk 4. Trying to normalize breastfeeding 5. Educate themselves on a more beneficial natural way for baby food** |  |
|  |  | **ActCOVID-ContactTracingorTesting** | **This code describes the switch to contact tracing and testing to address COVID-19** | **1. Focus on contact tracing from public health experts and tribal health organizations  2. Staff helping IHS with testing in the community  3. Public health efforts shifting to contact tracing and testing to limit the spread of the virus in these communities, particularly among vulnerable population groups** |  |
|  |  | **ActCOVID-WaterSanitation** | **This code describes dealing with water sanitation as a main issue emerging from COVID-19** | **1. Build water sanitation stations because most communities don't have running water 2. Need water to stay safe during COVID by washing hands frequently 3. Might not think that washing hands is a priority when a set amount of water has been designated for showering and washing dishes 4. Portable water stations that can be easily used by community members** |  |
|  |  | **ActCOVID-CommStaffInvolv** | **This code describes involving the community and staff in efforts to mitigate the overall impact of COVID-19** | **1. Having a survey to make sure that the community wanted the RCL program 2. Wanting to make sure that both staff and community feel safe 3. Wanting staff to feel that they're able to do their work without feeling that they're at risk** |  |
| **Planning for future programs during COVID-19** | **Pertain to the planning of the delivery of future sexual health programs to Native youth, and the possibility of switching programs to the online platform** | **PlanningFutureProg-ChallengesOnline** | **This code describes the main challenges of going online when creating and adapting sexual health programs in the nearby future** | **1. Fear that people will not accept the online version 2.Challenge is that people are trying to put programs hastily together 3. Programs not meeting the needs of young people 4. Very easy to do harm with young people when programming unintentionally** | **Question #8: *How will you be planning for the delivery of future sexual health programs in the midst of the ongoing COVID-19 pandemic?*** |
|  |  | **PlanningFutureProg-Feedback** | **This code describes the need for continuous feedback to ensure the success of current and future online sexual health programs** | **1. People need to focus on collecting regular feedback 2. Feedback lesson by lesson or session by session 3. Need to be asking young people what they like, what they would change to meet their needs 4. Be thoughtful and conscious about how we are putting programs together to support young people 5. Important to collect youth's feedback since the online platform is completely new and program developers and implementers need to ensure that they are meeting youth's needs** |  |
|  |  | **PlanningFutureProg-GuideDevelopment** | **This code describes the development of a guide to provide resources and steps for future program developers and implementers to follow** | **1. Developing a virtual adaptation guide for people to adapt programs with care 2. Transition to the online platform with thoughtfulness 3. Guide is encouraging communities to conduct a quick needs assessment 4. Get a sense of the challenges and facilitators in the community** |  |
|  |  | **PlanningFutureProg-SocialMedia** | **This code describes the importance of incorporating social media in the process of developing or creating online programs for Native youth, particularly for recruitment purposes** | **1. Get to know platforms and social media used by youth 2. Social media platforms can help with adapting the delivery of programs and recruitment** |  |
|  |  | **PlanningFutureProg-Process** | **This code describes the detailed and timely process needed to develop and implement an online curriculum for Native youth** | **1. Curriculum is one aspect, and implementation is another 2. Recruitment, retention, and evaluation should be tailored to be done online 3. Not as easy as slapping up program 4. Be thoughtful and conscious about how we are putting programs together to support young people** |  |
|  |  | **PlanningFutureProg-Training** | **This code describes the need for continuous training to make sure that all program developers are knowledgeable about the different social media platforms, the technological tools, and the management of websites** | **1. Two full days of trainings for virtual adaptation on how to implement We R Native curriculum 2. 80% rural sites participating in the training 3. Facilitators learning about holistic approach and management of the We R Native website 4. Educators provided with tools and resources on how to deliver and implement curricula virtually 5. Utilizing resources through multimedia 6. Technology education curve 7. Learning how to zoom one-on-one 8. Working with youth who don't know how to use zoom 9. Making it fun for youth on the screen 10. Training is one of the key factors ensuring the success of the program** |  |
|  |  | **PlanningFutureProg-Recruitinperson** | **This code describes the effectiveness of the in-person recruitment process in Native communities** | **1. Success in recruiting people when done in person 2. Social media is effective but in-person recruitment strategies such as face-to-face, going to tabling events, going to conferences, and going door to door are more effective 3. Even if programs convert to the online platform, an in-person component is still needed to engage people and get them excited about the program 4. Native communities relate more to in-person recruitment strategies as they feel a connection toward the educators of the program** |  |
|  |  | **PlanningFutureProg-RecruitStrategies** | **This code describes the need to be diligent when making flyers to make sure that the needed information is being communicated to the target population** | **1. A lot of writing in a flyer that no one wants to read 2. Diligence in making flyers because that what's being communicated to the people 3. Making sure people know what they are signing up for 4. Focusing more on word of mouth 5. There's a lot of information to include but sometimes not exciting for flyers** |  |
| **Reaching Youth through social media** | **Pertain to the utilization of effective social media platforms to reach youth online for recruitment and retention purposes** | **ReachYouthOnline-SocialMediaPlatform** | **This code describes the diverse social media platforms that were seen effective to reach youth** | **1. Young people are now going on Facebook to get more information about things like their programs and stuff 2. Facebook as an easy tool today 3. Success with Instagram 4. Youtube as one of the effective platforms 5. Schools are communicating with youth through emails, so they are checking emails more frequently which is a good and easy mechanism 6. Facilitators have been using their own social media platforms to reach out to youth and families in their communities 7. Getting the word out to youth and parents through emails 8. Different social media platforms have been used to try and reach out to Native youth. The most effective platforms have been Facebook, Instagram, and Youtube** | **Question #9&10: *How have you been reaching youth online since the start of the pandemic? In what way did the various social media platforms seem to be effective?*** |
|  |  | **ReachYouthOnline-In-Person** | **This code describes the continuation in using in-person recruitment methods alongside social media as it remains one of the effective methods for recruitment in Native communities** | **1. Some communities are going door-to-door for outreach to find youth that are disconnected or not online 2. For "We R Native" in Michigan, student handouts are printed and delivered to the doorstep of young people 3. Youth will then have handouts, can call in, or can be sent the powerpoint slides 4. Old school to go door-to-door but it's working 5. Flyers for recruitment is one of the effective ways 6. Put flyers in places that Native people visit such as a bulletin board in a primary building 7. Good tool for getting the word out** |  |
|  |  | **ReachYouthOnline-TrackReach** | **This code describes the strategies used to track reach of the program among Native youth and adjust channels used accordingly** | **1. Looking at channel analytics to assess reach and impressions 2. Track reach and adjust channels with messages 3. Youth health tech survey every couple of years 4. Better understand user preferences for accessing health information 5. Channels youth feel comfortable utilizing 6. Leverage existing celebrities or content creators 7. Making presence on channel stronger 8. Shift in channels; youth are not going to websites for information but using other social media channels 9. Thinking more creatively about other channels like Tik Tok 10. Analytics helping since able to garner feedback from a distance** |  |
|  |  | **ReachYouthOnline-NewPlatforms** | **This code describes the search for new platforms to improve reach and increase interaction with youth** | **1. Exploring other effective platforms like Podcasts 2. It might be a new thing for Native youth to engage in podcasts 3. Trying to catch-up with technology shifting immediately 4. Tick Tok not effective in some communities 5. Still figuring out how to create videos on Tik Tok for RCL 6. Social media turning from a connection platform to an educational platform** |  |
|  |  | **ReachYouthOnline-NewNorm** | **This code describes the shift to a new technological norm in the delivery of programs** | **1. Questioning how to shift as a team and adapt 2. How to create online virtual gatherings and online conferences 3. How to engage youth online 4. Zoom culture now where some people prefer not to engage 5. Tone that you start off with on zoom can make it informal and fun 6. Getting creative and going online has been the norm now 7. Figuring out how to balance the mental health of technology 8. Stop the scrolling market in a way where this is exciting, like sexual health is vibrant and bright and something I want to engage in 9. Shifting the marketing strategy as a team 10. How to make material more live and more appealing for Native youth** |  |
|  |  | **ReachYouthOnline-InclusivePlatform** | **This code describes the openness of the platforms developed for youth and the inclusiveness aspect, which lead to an increased participation from the LGBTQ youth** | **1. A lot of Native youth are opening up about sexual health 2. Pushing LGBTQ platform, body image platform, and sexual health platform into a more positive light** |  |
|  |  | **ReachYouthOnline-YouthAmbassador** | **This code describes the need for youth ambassadors to help with the development of future programs and provide recommendations for recruitment and engagement purposes** | **1. Hired a youth ambassador who completed the RCL program 2. Youth ambassador knowledgeable about RCL lessons 3. Helping them with curriculum by giving them a youthful gaze 4. Helping them with recruitment and making content more exciting 5. Helping them with tick tok segment 6. Providing them with an outside perspective regarding youth activity preferences, interactive tools of interest, and modifications that are needed to improve the overall reach and efficacy of the program** |  |
| **Adapting/Creating Online Sexual Health Programs** | **Pertain to the adaptation/creation process when adapting or developing online sexual health programs for Native youth** | **AdaptCreateOnlineProg-Training** | **This code describes the need for continuous training during the adaptation or creation of online sexual health programs for Native youth** | **1. Trainings, going through all steps, needs assessment, looking at logistics 2. 12-hour trainings on how to adapt We R Native 3. Reminding people that they still need to go through the same steps when creating a program in the adaptation process 4. Don't shortchange or skip steps in hte planning or preparation process 5. Reminding people that even if they need to shrink the duration of the needs assessment, it still needs to be done 6. Needs assessment to understand how the school district is doing, what are the implementation partners doing, and what platforms are being used 7. Look at logistics because every program is different 8. Training, retaining all staff, new curriculum, and new virtual protocols and procedures** | **Question #11: *Tell me about your experience in adapting or creating sexual health education programs for Native youth using online platforms. Tell me from start to finish how you went about it.*** |
|  |  | **AdaptCreateOnlineProg-Platformoptions** | **This code describes the availability of multiple platform options to reach youth and increase interaction in game-based activities** | **1. Using google classroom in some schools since they can put entire program through this platform when needed 2. Instructional methods like quizzes and worksheets are easliy adapted to the online platform 3. If it's game-based activity, it can be challenging to adapt to the online platform 4. Switching the program model, some of them are already online like We R Native 5. Zoom, GoToMeeting, and Webex as posting platforms 6. Platform for young people to access files at their own time 7. Introducing different platforms and showing people the difference between zoom versus google jamboard 8. Can use different programs in conjunction with each other to make a program more interactive and effective 9. Don't want people to just put powerpoint slides and lecture for hours** |  |
|  |  | **AdaptCreateOnlineProg-ResponseNeeds** | **This code describes the need to respond to community needs whenever developing or adapting programs** | **1. Being responsive to family needs 2. Making sure that the pedagological methods align with the interactive methods 3. Adapting the curriculm to meet the community needs in a virtual format** |  |
|  |  | **AdaptCreateOnlineProg-ThinkingLongTerm** | **This code describes the need to think about the online platform as a long-term solution rather than a temporary one** | **1. One of the biggest problems is that people have been thinking repeatedly that COVID is going to stay for a month or two 2. If you think that you're only going to do online implementation one time, you won't put a lot of effort into it 3. If you're planning to implement with young people for the next year and a half, it's probably going to be online 4. Reflective piece as a long-term thing 5. Thinking about it as something for the year and a half to two years 6. Need to stop acting that COVID will be over in a month or two 7. Accommodate programs to meet the online needs of the community in a culturally tailored and appropriate way** |  |
|  |  | **AdaptCreateOnlineProg-Monitoring** | **This code describes the need for continuous monitoring of newly adapted programs for quality improvement purposes** | **1. Developing group cohesion online 2. Keeping interactive features, thinking about doing them in a new way, or substituting them for another interactive feature 3. Monitoring and quality improvement with continuous feedback 4. Fidelity monitoring 5. Reaching the community through community feedback 6. Survey will highlight ways to reach the community and what ideas would they have 7. Whether virtual program is feasible from a community point of view 8. Doing iterative quality control and improvement during piloting 9. Implementation forms used by youth and parents** |  |
|  |  | **AdaptCreateOnlineProg-WorkingTogether** | **This code describes the need to work together as a team to increase visibility and recognition of the program among community members** | **1. Team members working on differetn projects meet once a week 2. Take a look at what needs recognition, what needs visibility, what needs to be elevated in our communication, dissemination strategies 3. Teams are personally tied to their communities and get a pulse of what is trending 4. Better-informed approach of what is resonating 5. Session summary forms filled by staff 6. Weekly ongoing debrief sessions with staff to go over lessons learned from youth 7. Sharing ideas for changing program and making it better 8. First step is getting team buy-in 9. Make sure that team feels that everything in the program make sense to the community** |  |
|  |  | **AdaptCreateOnlineProg-CulturallyTailored** | **This code describes the need to tailor messaging and social media channels to Native youth** | **1. Trying to tailor messaging and channels to specific demographics (youth, young adults, parents, and educators) 2. Trying to make it attractive through visuals 3. Drawing folks through newsletters or posts 4. One thing resonates across the board is that "Natives like Natives" 5. Natives want to see themselves in the curriculum products, videos, messages, and themes 6. Tagline of Native culture is prevention** |  |
|  |  | **AdaptCreateOnlineProg-Collaboration** | **This code describes the need for continuous collaboration between schools and other public health entities for the successful delivery of sexual health programs for Native youth** | **1. Collaborations happening between community health or behavioral health departments and local schools 2. Two entities coming together to offer sexual health classes 3. Could be done for the fact that this is a tribal school on a tribal land that's looking for tribal self in sexual health 4. Still new in this infancy because of Healthy Native Youth and certain projects 5. Federal response with a curriculum and a list that can be utilized for some programs** |  |
|  |  | **AdaptCreateOnlineProg-HolisticApproach** | **This code describes the need to take a holistic approach to sexual health since different aspects of Native youth health have been affected as a result of the pandemic** | **1. More holistic approach to sexual health by including mental health, social health, cultural health, and physical health 2. Adults learn with the youth in a more modern context 3. Neutralizing any fear that adults have 4. Outside noise that render sex as a taboo** |  |
| **Benefits & Barriers of Switching to the Online Platform** | **Pertain to the benefits and barriers associated with switching sexual health programs online** | **Benefits&BarriersOnline-Flexibility** | **This code describes the benefits of having a flexible online sexual health program that can be tailored and changed** | **1. Building curricula in a variety of settings 2. Person delivering the curriculum might wear a variety of hats (teacher, health educator, parent) 3. Responding to the needs of the community through these programs 4. Exciting for youth to have tech-savvy programs 5. Flexibility is needed since technology is always advancing and things are continuously evolving** | **Question #12: *What are the main benefits and barriers for switching programs to an online version in Native communities?*** |
|  |  | **Benefits&BarriersOnline-TechSavvy** | **This code describes the need for tech savvy programs to grab the attention of Native youth living in a technological era** | **1. Don't want to feel left behind with an old program 2. In need of something new and tech savvy 3. New technologies to make it more exciting for youth** |  |
|  |  | **Benefits&BarriersOnline-Human Component** | **This code describes the barrier of losing the human component when adapting in-person programs to the online platform** | **1. Create safe spaces for reproductive health knowledge 2. Creating relationships is different online 3. Youth are missing out on learning in larger classroom settings 4. In-person component is part of Native cultural values** |  |
|  |  | **Benefits&BarriersOnline-UnsafeHouseholds** | **This code describes the challenge of having unsafe households when delivering sexual health programs** | **1. Living in a household where participating in a sexual health program is not accepted 2. Depends on young people's situation at home 3. Parents are typically not open-minded about sexual health programs in rural areas** |  |
