# Supplementary material for: The Impact of COVID-19 on the Delivery of Educational Programs in Native American Communities: Qualitative Study
Source: JMIR Form Res. 2022 Apr 11;6(4):e32325. doi: 10.2196/32325 (PMC9004623; doi:10.2196/32325)
Supplement: Multimedia Appendix 3 [file formative_v6i4e32325_app3.docx]

Appendix 3. Quotations for Themes and their Relevant Sub-Themes

| **Themes** | **Sub-themes** | **Principle Verbatims** |
| --- | --- | --- |
| **Competing Community Priorities during COVID-19** | **Food Security & Water Sanitation Measures** | **"I mean, the food insecurity, I feel like we never hear about that on the news. But, um, the food insecurity is a major, major issue, like meeting the basic needs of households has been a primary thing that I've been hearing from, from Native communities and, and others, you know, that they're, they're either doing contact tracing, or they're doing food relief" (Int1)** |
|  |  | **"So, with these care packages that they created was just a combination of like, different like supplies. But I think in terms of security, one of the big challenges was food formula for like, babies and infants and such, toddlers, probably. I know, that was a really big challenge. And it was a big challenge before in communities because of I think just trying to normalize breastfeeding for a really long time. And then when COVID hit, I think a lot of people now sort of under realize, like there wasn't enough formula to get there wasn't enough food, there was not enough wipes. So, then they're like, oh, let's try this breastfeeding thing. And low and behold, you know, they did find that it was more of a natural way and helpful" (Int2)** |
|  |  | **"So I think too, we did learn from another site as a team that when folks were engaging other youth and other activities, they gave out like grubhub gift cards versus doing like Amazon gift cards, or online gift cards. So, I think that may have helped a little bit better" (Int2)** |
|  |  | **"But our center really quickly pivoted to having like to addressing food insecurity as well as COVID related contact tracing COVID testing sites as well. So we give out wellness boxes and both of the communities that are that have RCL, where families that don't have enough food or don't have adequate access to food can get these wellness boxes that are that have a lot of food" (Int3)** |
|  |  | **"We've also built water sanitation stations because a lot of the communities in our that we work with don't always have running water. So, this to be safe during COVID. It's super important right to wash your hands and if you don't have access to running water or you only have some if you have a set amount of water, right? And you've designated that for showering and washing dishes, you may not think that washing hands that Is your priority if you only have a certain amount of water" (Int3)** |
|  |  | **"And we've also been building hand washing stations. So like I was saying earlier, people don't have running water. And so you can ask people to wash their hands, if they don't have running water, they need to save that water to drink and to clean themselves and to, you know, cook their food. So we've been creating hand washing stations that we've also been delivering to our various partners" (Int4)** |
|  |  | **"In some of the communities, you have homes where people don't have access to running water, they don't have electricity, you have a lot of families living together in close quarters, which make things like hand washing and social distancing, and isolation and quarantine almost impossible" (Int4)** |
|  |  | **"We've been doing a lot of food delivery. So we've been getting a lot of donations from different organizations and soliciting donations. And then, you know, these communities where we work are huge, and it takes hours and hours to get to some of the neighborhoods, you know, in some of the cities, so we'll, so our team does a lot of driving to deliver food boxes" (Int4)** |
|  | **Financial Hardship** | **"A lot of young people here are having to take jobs as part of like, meaning to contribute when their parents have lost their jobs, which throws their ability to participate in programs and even their education into peril" (Int1)** |
|  |  | **"And so some of the challenges that were already existing in their communities as far as like, economic challenges, lack of access to resources and some of the rural areas those young people have whose families have been impacted by losing jobs or things like that" (Int1)** |
|  |  | **"They have to deal with food insecurity, and, like, unemployment, so the kids have to help their parents, that is a national dilemma, right, you know, everybody sort of, kind of everything sort of falling away. But the basics, you know, and, you know, it's an unfortunate thing about this pandemic, is it's really just shedding a light on long standing inequities, and deep seated inequities between groups in this country. And you know, it's really just shining light on that. And hopefully, some good will come of it" (Int4)** |
|  |  | **"And a lot of the students some of them might be home, like back in their hometowns. So a few of them, I think they're still in the town of Tucson, but a lot of them probably have stayed home with their families. So but they have other responsibilities with their families, too" (Int5)** |
|  |  | **"And then that coupled with tribes being on furlough, people losing their jobs. Yeah, people, yeah, communities losing their, their youth center, due to having to re-allocate resources to other projects. So it's, yeah, it's been a pretty big impact" (Int6)** |
|  | **Mental Health Impact** | **"And most of our tribes are struggling to support the mental health and well-being of the people they serve. Um, we're already seeing that on both, even here in the Southwest, we're seeing it so that that lack of stability is really hard for young people the like, not knowing what to expect, is very difficult for them to cope with" (Int1)** |
|  |  | **"And I think our we're at real risk of like a widespread mental health crisis for young people, for teens, particularly. So we cannot, we cannot not address that, like young people's lives are at stake when it when it comes to a time like this. So. So that would be like mental health piece is one of my number one thing to do would be to, like, build competence in, in how to do this and be really, really thoughtful about how you plan and prepare and deliver your programming" (Int1)** |
|  |  | **"And so I got to see firsthand and visit with some folks and their rural location and with the youth that I spoke to, you know, I think, like the rest of like, all of us a lot of stress, a lot of anxiety, a lot of worry, a lot of unknown. You know, it's… mental health is definitely an issue" (Int2)** |
|  |  | **"So that, on a community level is devastating. We've lost a lot of elders who are just carrying a lot of wisdom and knowledge about their culture and their beliefs. And in that sense, from a from a cultural point of view, it's been devastating. From a human loss point of view, it's been devastating" (Int3)** |
|  |  | **"And it's a process and things are changing all the time, you know, the, the pandemic seemed to be under control to some degree, and now it's ticking back up. And that changes the landscape, you know, it's going to change, people's willingness to engage online, I'm going to change their thoughts and hopes about the next few months. So it's going to change their mental health and all those things. So it's all I would say, I would say, I don't think I know all the answers" (Int4)** |
|  |  | **"But I think the youth really need us to be there. They all say where they're fine. They're doing okay. But I think the impact of this pandemic can is really on the mental health. A lot of people are not fine. And this is really a big change for the students" (Int5)** |
|  |  | **"So I think the challenges of moving from the physical space to a virtual space is that disconnection from being around folks physically, I think that's one that I hear often is that, you know, with tribal communities in particular, at least from my experience, we've got a big hug of people. I think the mental health impacts for that are going to be pretty huge in the next decade or so. Yeah, so that I that's, I think that the major piece there" (Int6)** |
|  | **Focusing on COVID-19 Response in Clinics and Centers** | **"So a lot of public health clinics have shifted their focus away from some of the core of public health, which is, you know, contraception, they have shifted away from that, because they're, they're having to do COVID. So yeah, quite a bit in even the non-clinical side, like, even in youth programs, a lot of staff have been reassigned to COVID response and contact tracing. And so yeah, a lot of those services are not as accessible" (Int1)** |
|  |  | **"And then one more thing, too, is I do know, there has been a lot of like hesitancy in terms of going into different clinical systems in terms of sexual health care. So getting those like regular checkups, a lot of folks that are a little hesitant to go into the, to their local tribes or communities just because of like, you know, the hospital, and they don't want to get COVID because, again, in some communities, they only have one health clinic space. And so those, you know, probably too, where maybe some patients COVID positive patients go to, so I think those are some things that I think travel communities, but also learning to navigate" (Int2)** |
|  |  | **"Some of these clinics were had staging areas outside of their clinics in order to be able to do intake, whether it was a potential COVID patient, or what have you. And in during the wildfires, the air quality was so severely unhealthy, they just shut everything down. So, um, access to services is still an issue, but you know, trying to communicate to youth that they have this option, and the conduit for those folks are facilitators are there" (Int2)** |
|  |  | **"And through our partnerships with IHS, we've also been asked to help them with other COVID related efforts. So, some of that, but that all that work is new, and not necessarily what our team was doing prior to COVID. And it's constantly changing, and in flux that both of both the communities that we work with the most closely, there have been weeks where we have our team back, and it's almost as if you know, it's not COVID, staff attendance and availability, but then, you know, the surge and we lose some of our staff members to COVID related for necessary COVID related work" (Int3)** |
|  |  | **"But our center really quickly pivoted to having like to addressing COVID related contact tracing COVID testing sites as well. So we have like a couple of testing sites like regularly that happened in the community where we, our staff are the ones that help, IHS, or we ourselves, set them up to do testing in the community, we have what we call them, and then we help with contact tracing as well" (Int3)** |
|  |  | **"Yeah, I mean, our center really pivoted to be like public health responders, you know, we we, again, knowing that what we know about the communities where we work and all of our colleagues that we've had for, you know, 40 years, we knew what how this was going to impact them. So we really, you know, we have a lot of infectious disease research experts within our center and also within the school. And so we look to them to really create like an on the ground plan. So a lot of our staff, you know, were trained in contact tracing. And in COVID, testing, they've been doing that they've been part of contact tracing teams, they've been part of, you know, swabbing and testing teams and mobile clinics" (Int4)** |
|  |  | **"I think they're slowly going back to opening up their health centers, but I think scheduling a lot of is virtual zoom schedules. The WIC department is, I think still not seeing their patients and their families face to face yet so I'm not sure how it's going to be looking by the beginning of next year" (Int5)** |
|  |  | **"So it's telehealth. And, yeah, so there's been recommendations for youth to you know, get in touch with their telehealth. And then we don't know how it's going, how that's actually translating, and how that's actually working if it's being successful or not. So yeah, there's been content that we're creating around how to access sexual health resources. But yeah, from tribe to tribe, it's very different" (Int6)** |
|  | **Sexual health as a Secondary Concern** | **"So a lot of our program has had to shift from that hyena thinking about like the Maslow's hierarchy of needs, right, we're shifting away from the sex ed aspect all together, and we're doing, you know, our teams are doing food deliveries and supply deliveries, PPE stuff" (Int1)** |
|  |  | **"And now we're seeing a federal response, saying here is a curriculum, a list that can be utilized for some of these programs. I think what's interesting is that it's just not the silo of physical sexual health. It's more holistic, mental health, social health, cultural health and physical health" (Int2)** |
|  |  | **"But reproductive health curriculum is considered extra, and therefore, sometimes gets cut not all schools, but excuse me, in some schools. As far as that that's just been challenging" (Int3)** |
|  |  | **"But we had some interested people who we kind of kept like a list of. And so those participants actually were the ones that were interested in RCL. And they kind of felt that despite the pandemic, they still wanted to take part. And that kind of was our motivation to say, okay, good, the community is still interested in this, they still want this program, despite everything that's going on. Yeah, that was, I think, not surprising, because we know that we didn't think people wouldn’t be interested. But we just knew that people's priorities may have shifted" (Int3)** |
|  |  | **"And maybe people are so worried about other things that sexual health is probably not a priority for them" (Int5)** |
|  |  | **"It's definitely been placed on the back burner" (Int6)** |
|  |  | **"You know, working at the tribal center, its COVID response, and so it was prioritizing needs, and the needs weren't sexual health, per se, it was making sure that everyone was safe that their families receive. So it was refocusing the sexual health content to be mostly around mental health focus, and relationship building. So adapting whatever topical area might have been included in a sexual health curriculum, say healthy relationships, and reframing that message to be in a more informal space that allowed to build relationships. That was a huge impact" (Int6)** |
| **Moving to Web-based Programming: Skills, Training, Support** | **Adaptation of Programs to the Online Platform** | **"And so, writing a grant from the perspective of like, we don't, you know, it'd be new, we didn't know how to plan and how to write, you know, for that. So, we just wrote it as if we were implementing as normal and figured we would figure out the adaptation process later. And so, so that's been kind of a challenge now that we've been awarded, we're trying to figure out how to how to implement virtually how to coordinate with a school who's doing blended learning and meet their needs, as well" (Int1)** |
|  |  | **"It can be very quick to open up a zoom account and get started on zoom. Right? You can do it in minutes. But that doesn't mean that you're prepared to implement a program online. And so. So, I think that one of the challenges is that people are going to, I think, initially hastily put their programs together, and they're not going to be good. And they're not going to actually meet the needs of young people" (Int1)** |
|  |  | **"And so, you know, very quickly, we had to start adopting our own programming for virtual settings. And, you know, kind of identify which components of our work we're going to get continue happening in community settings, because most of our tribal clinics closed, our tribal schools closed, public schools closed. So, kind of being in touch with our tribal health educators and our clinic staff to see which services and programs were continuing, what was being paused, what priorities they had for moving forward and trying to respond to those" (Int2)** |
|  |  | **"So, we cancelled that camp and quickly we had to figure out how to do RCL. So in in light of COVID, we decided that it may make sense to try a pilot program of RCL being conducted virtually. We have not done ourselves in a virtual format before, we've been used, working with RCL for quite a while now several years, if not more. But it's never been done virtually. So, this was brand new for us. And it was… it's a program that requires a lot of in-person activities" (Int3)** |
|  |  | **"For the current way that we implement the respecting the Circle of Life program, it is with federal funds, but it's, there's more flexibility with this particular federal funder, like there, they were really encouraging of us to try a virtual implementation, which we've been doing for the last few months, we've been trying to deliver the program via zoom. And that's been, you know, a real learning process. But, um, you know, really, most of our programs are pretty much halted with the exception of respecting the circle of life, which you've been trying to do virtually" (Int4)** |
|  |  | **"Yeah, I think it was just a huge delay in trying to figure out what to do in terms of adapting content. So some of the content that we work with pre-COVID was in an online platform. And so obviously, that translated easier. But even then, they most folks were still meeting with youth in some capacity physically, even though it might have been, you know, multimedia. Yeah. Technology that they were using. So yeah, lots of adaptations" (Int6)** |
|  | **Lack of Sufficient Time and Staff Support in Stressful Adaptation Process** | **"And they're, like requesting it, they're begging for support. And it's all over the country. I mean, the level of support that people need right now is substantial, I'm working like seven days a week, for weeks on end, like during, during this crisis, because the level of support that communities need in order to adapt and to try to function at all, in supporting young people's needs is substantial" (Int1)** |
|  |  | **"And so yeah, that has been an enormous challenge, because it's not like we can say, Oh, just shift everything to these online tools. We first have to teach the tools and how to use them and get some basic skills under their belt and then start to look at, you know, the other piece that comes after that is that I'm a curriculum specialist" (Int1)** |
|  |  | **"So, although some of these were presented in person, there was a lot of adaptation to go online, and what that look like, so a lot of trial and error, a lot of challenges and a lot of different avenues of communication and having to be also creative, and also still innovative too (Int2)** |
|  |  | **"Secondly, was how to coordinate our schedules and utilize a virtual platform such as zoom, and become zoom savvy for us as professionals, and I can tell you, I think we've talked about it, we've been more busy now in a virtual setting than we have when we were in the office" (Int2)** |
|  |  | **"We also had time constraints because we wanted to get this up and running and stay fairly focused, and to do everything all at once, it just wouldn't be feasible and also it may not create kind of a cohesiveness" (Int3)** |
|  |  | **"I know a lot of tribal employees were told to work from home, which is a lot of them don't have the internet to work from home. So I think a lot of that was trying to adapt to it and try to find resources that can assist them" (Int5)** |
|  |  | **"So, we're sort of like, we keep telling people, we're like pulling the curtain back a little bit to show you the background of what this looks like to run a session like this, because it helps them build those skills and understand like, oh, here's how you, you know, use a quiz or a poll or something on zoom or another online platform. And yeah, just introducing some of those basic tools. So, we've been doing that. And then, we're also writing a guide that we've been working on for like six weeks. But it's, it's tough, because we want it to be robust enough to actually support communities in that adaptation process" (Int1)** |
|  |  | **"Trainings are great. We've been doing a lot of trainings and a lot of recorded sessions on YouTube. That's been a big, I think, a facilitator for us in our programming" (Int2)** |
|  |  | **"The second thing about this, though, is a technology education curve, how including myself in that group, you know, learning how to how to zoom, you know, zoom one-on-one, and then working with youth who may not know zoom, they only know FaceTime, you know, or Facebook Live" (Int2)** |
|  |  | **"So, we had to incorporate an entire tech training on how to use zoom and how to use like the internet to do a lot of things. And I mentioned our team, our paraprofessionals, which means they're from the community, but they may not always have all of the skills. So, for us training is really important to make sure that we're helping our team members kind of reach their best potential, so they can do all of these things" (Int3)** |
|  |  | **"And so we have a really strong curriculum team that does all the trainings with the, with the facilitators, and then we have a very robust system for ensuring that they feel ready to go and so that just includes a ton of role playing with each other with us. There's an exam that they have to take at the end. There's also like a competency checklist that they take at the end just to ensure that they understand the content and feel prepared for the delivery, but they also understand, you know, how to be proficient with zoom and to troubleshoot different tech challenges that will come up along the way. You know, so I think that I think that we actually are doing well" (Int4)** |
|  |  | **"I think it was, say it was both kind of learning how to get in information and providing it in a virtual setting, a lot of it was changes because all of our, our work, we do a lot of face to face training" (Int5)** |
|  |  | **"So holding trainings for the adults to try out breakout rooms, and try the whiteboard and the annotation features and trying out other apps, you know, that make online learning a bit more engaging" (Int6)** |
|  | **Youth Missing One-on-One Connection** | **"But I think even if we are able to convert our programs to being fully virtual, I think there is still going to have to be some in person components to really like, engage people and get them excited about the program" (Int3)** |
|  |  | **"So we have had, we have had some zoom sessions, where there are, you know, siblings together with their parent, and there's a couple kids, but their original program, you know, kids are in groups, and they're doing all these fun activities, and they're laughing, and they're really engaged. It's very much like an interactive process. And so, you know, some of the activities that were like that originally that we knew we couldn't do a resume we either took out or kind of modified" (Int4)** |
|  |  | **"Yeah, so that was the I think one of the impacts was a lot of the students were required to meet, like, they had like a little house on campus. So that was kind of like, their place to hang out together. And it was like a kitchen for them. So sometimes they would have food there. And especially when you're a college student, you know, sometimes you don't have the money. Yeah, but since that pandemic happened, a lot of it is virtual. So some of the students are actually probably missing that one on one connection" (Int5)** |
|  |  | **"But for tribal communities, the in-person part is such a major piece. And I don't see that changing in any capacity, I do think that the virtual piece will be really amped up, and I think it will be smoothed over quite a bit" (Int6)** |
| **Recruiting Youth** | **Using Social Media Platforms for Youth Outreach** | **"We've been using Instagram quite a bit. It's been I've been kind of waiting for Instagram to go out of fashion with young people. And so far, it has not. I've been surprised. We tried on a project I'm working on, we tried to get young people to do some things on Tick tock, and they were like, oh, we'd rather do it on Instagram" (Int1)** |
|  |  | **"So, I think that they, like previously, young people wouldn't be caught dead on Facebook. And now they are going on to Facebook a little more to get information about things like particularly their programs and stuff" (Int1)** |
|  |  | **"So, we run kind of a multimedia Health Resource for Native youth that includes the website, Facebook, Instagram, Twitter, and then you know, so we can kind of see the analytics on those channels, and what sort of messages are getting the best reach and impressions" (Int2)** |
|  |  | **"Our facilitators themselves have used their personal social media to reach out to youth and families that they know in their communities, so they're able to post things on their Facebook page, or on like, like, there's yard sale pages and things like that, that are more community focused. So, they are able to post there. Facebook has been really helpful. I think that's the main tool we've used. They also I think, had a little bit of success with Instagram, we're trying to see if we can get to tick tock started" (Int3)** |
|  |  | **"We use Facebook and Instagram; we've we haven't quite gotten into tik tok yet" (Int4)** |
|  |  | **"We just have Facebook right now" (Int5)** |
|  |  | **"Yeah, um, so all of the social media channels, mostly focused on Instagram, YouTube, as the primary focus, and then Facebook, mostly for getting content to the older, the adults" (Int6)** |
|  |  | **"But generally, every year or so we'll do a tech survey, just to make sure the platforms that we're using are current and the content that we're posting is relevant. And yeah, so that's why we're focusing on Instagram in particular" (Int6)** |
|  | **Differences in Online Program Youth Participation Rate** | **"Yeah, there’s definitely… the recruitment and the retention in a virtual setting has is… versus an in-person implementation, yes, there is a dramatic difference. Only because of the fact that you know, that we're trying to ourselves as an organization, and educators trying to find resources and tools that they can implement, if they were to implement a curricula, trying to adapt that. The upside that we're seeing is an uptick in our social messaging for youth. That's a very popular resource" (Int2)** |
|  |  | **"We did see a little slight decline since the virtual setting for schools that some of the kids didn't come back. So we don't we don't know where they went" (Int5)** |
|  |  | **"Yeah. And the tribe in particular that I'm thinking about that we are working pretty closely with the sexual health curriculum, their participation had dropped off pretty dramatically, and most of the youth that they were seeing was only on a mental health counseling basis" (Int6)** |
|  |  | **"But we, you know, yeah, part of it is, I think the programs that are going to be successful at recruitment is programs that are operating through a school, it's much harder already, like some of my teams in Michigan have been trying to get the young people together, and they're just now getting to the point. And it's because school has created this, like, sort of organizing opportunity, like kids are having a more structured day than what they have over the summer. And in the early days of the shutdown" (Int1)** |
|  |  | **"And, you know, kind of identify which components of our work we're going to get continue happening in community settings, because most of our tribal clinics closed, our tribal schools closed, public schools closed. So, kind of being in touch with our tribal health educators and our clinic staff to see which services and programs were continuing, what was being paused, what priorities they had for moving forward and trying to respond to those" (Int2)** |
|  |  | **"And our team kind of talked to schools, they talked to a superintendent, they were trying to figure out how schools are going to be doing things even though it was the summer, their summer schools. So, we wanted to see what kind of how that would make sense in the community. Another thing to consider is that site is very rural, and very remote and very impoverished. And if we did this program virtually, would that be a barrier, right, having access is crucial and important, especially in a virtual program. So that was one of the things that we had asked our staff to kind of look into a bit more to see, you know, there's different ways that we can make sure you get internet access, is internet going to be a problem that comes up recruiting" (Int3)** |
|  |  | **"Yeah, I think, I think with schools closing, you know, for programs that are offered in schools, you know, that made it impossible" (Int4)** |
|  |  | **"Some of them were talking about the impact of schools, not being able to go in schools to educate. And then that barrier, and also kind of a lot of the sexual health education is one on one, I guess. And I don't know that people are more open to be talking one on one to somebody about sexual health. But more the outreach has slowed down. Now they have to find a different method of outreaching" (Int5)** |
|  | **Distribution of Flyers in Community Locations** | **"That's one of the effective ways. I mean, flyers in a place that people are still going so um, you know, in in some of the smaller Native communities like that, there may be like, people actually go and visit a bulletin board or they go and visit like something in a primary building. Or especially like, where things like, you know, the food pickups are happening, a flyer on a bulletin board or a flyer in a location like that can be, you know, a good tool for getting the word out" (Int1)** |
|  |  | **"I think there is still going to have to be some in person components to really like, engage people and get them excited about the program. Because I think also being in research there's you have to when you advertise things, right, it's a lot of information you have to make sure you include, which is all good, but it's not necessarily the most exciting like recruitment flyers" (Int3)** |
|  |  | **"We also do continue to distribute, you know, flyers and community locations, we put flyers on people's cars. We have some billboards, like electronic billboards, where we've bought out time for, you know, recruitment materials. So, you know, we're really just trying to work the channels that are existing in the community" (Int4)** |
|  |  | **"So and especially working with tribes, a lot of tribes like to travel out because a lot of them don't have the internet resources. So that was another impact was trying to find the communication source because a lot of it was is going to be email, but kind of creating flyers and resource lists was the other…was another thing" (Int5)** |
| **Challenges for Implementation in Household Environment** | **Challenge of Youth Program Participation from Home Environment** | **"The parents will say, oh, I'm going to put or sign up all four of my kids who are all in that age group to be in this class together, and they're going to all join. So, we weren't sure how that would work out. We weren't sure. Would youth just sign in from different devices? Because that makes polling and these interactive features really doable? Or will you if they're in the same house, if they're siblings, for example, or cousins, or relatives or you know, a germ unit that are all seeing each other regularly? Are they going to just sign in from one computer? Because then that throws some of the activities we planned, it changes them a little bit? Right?" (Int3)** |
|  |  | **"And we've all kind of heard this, like zoom fatigue is such a thing where you're just on video calls all day, and you're exhausted. So, we didn't want this to be one more thing that a kid has to do that it's something that they want to do" (Int3)** |
|  |  | **"And part of what is, what's nice about the camp model is the kids can kind of work through those nerves together. And there's like some fun games they play and things they can do to kind of practice things, some of the phrases and the words that are a little bit uncomfortable. And we do some of that in the virtual model. But it is it is more challenging when it's like a one on one situation versus like a fun group at a camp" (Int4)** |
|  |  | **"So but they have other responsibilities with their families, too. So right now I am mentoring one person. And my communication with her is very difficult, because she lives far in New Mexico so and she kind of has family that she has to watch. Since she's not in school down in Tucson. She's doing her virtual schooling from there. So that is a little challenge" (Int5)** |
|  |  | **"But you know, there's lots of need around, you know, with intergenerational families sharing technology. So you know, maybe the youth was assigned a computer that or you know, a tablet that did have data on it, but then that youth might have to be sharing it with siblings or the adults. And then also family schedules. So you know, if there's a family and this youth is supposed to be on a sexual health lesson, but then mom needs to go grocery shopping, just take all the kids with her" (Int6)** |
|  |  | **"So, for instance, young people that are living in homes that aren't safe, and that may be like participating in a sexual health program, in a household where that is not accepted. Whereas a young person could have previously like said, Oh, my after-school program is like, spirit club or something, when it was actually a sexual health program. Um, you know, now, if they're doing something at home, they are in a home where other people may see what they're doing and know what they're up to. And in homes that are not safe to do that, that can be a real barrier" (Int1)** |
|  |  | **"Even on clinical services, we're seeing that like, you know, pediatricians and doctors that are working in adolescent health, they're saying, like, I can't talk to, you know, these young people in a virtual visit in a safe way. Or we're having to ask them, like, is this is this a safe time to talk? You know, do you have privacy right now? Which is, you know, it's hard. We have previously, you would have asked the parents to just leave the leave the exam room, but yeah, so. So yeah, I think that it sort of like, it really depends on young people situation at home, and it's a lot more, there's a lot more room for young people to be in trouble for participating in the sexual health program. whereas previously, their parents wouldn't be there watching what's happening in the program. And parents typically are not as open minded about these issues, especially in Texas, and in you know, rural Michigan, conservative Michigan. These are not communities where it's easy to do sexual health and so, so yeah, I think that can be a real barrier as well" (Int1)** |
|  |  | **"I think, like I mentioned earlier, and I jokingly said it before, it kind of ropes back to this, is the adults learn right along with the youth in a more modern context, and neutralizing any fear. I think it's just the outside noise that makes it seem like it should be taboo. And it's really not, it's just, that's why with this generation through the technology and the conversation, they're taking it and owning it. Just the older folks, that's gotta let them learn and, and let them live. And we offer that guidance, but they make the choices" (Int2)** |
|  |  | **"And that last session is to hopefully increase communication between the youth and the parent. We also do a condom demonstration and talk about safe sex and all of these things with the parent and youth together" (Int3)** |
|  |  | **"As much as we love tech, on the other hand of it is like people connection, people interaction is so important, especially for creating safe spaces for Reproductive Health knowledge. And like creating this relationship between the user and the facilitator, the youth and other youth like youth are missing out on learning in larger classroom settings" (Int3)** |
|  |  | **"And the kind of like, the taboo of tribal communities, of talking about sexual health, and the embarrassment of condom use, and even taking one or knowing somebody is passing out that information" (Int5)** |
|  |  | **"Now, a lot of rural youth also have the challenge of that they don't have internet connection, stable internet connection, or high-speed internet, which is really shocking that it's 2020" (Int1)** |
|  |  | **"But we've also heard collectively, what, what the needs are. And, and that's pretty compelling from the field, infrastructure, weeds. You know, there is a digital divide out there. So not everybody can utilize a computer and a laptop or enroll America in most places. So that's been pretty compelling" (Int2)** |
|  |  | **"So that all to say that not all youth would have computers at home, not all youth would have internet access at home. And if we did this program virtually, would that be a barrier, right, having access is crucial and important, especially in a virtual program. And so, we definitely wanted to be mindful of that. So that was one of the things that we had asked our staff to kind of look into a bit more to see, you know, there's different ways that we can make sure you get internet access, is internet going to be a problem that comes up recruiting" (Int3)** |
|  |  | **"You have to make your program virtual, because how else would you reach these people? I mean, the challenge of it is, technology is not perfect. So, having like losing internet is something that you have to take into account, and you miss the human component, right" (Int3)** |
|  |  | **"So and especially working with tribes, a lot of tribes like to travel out because a lot of them don't have the internet resources" (Int5)** |
|  |  | **"Right now is the internet. That's the big thing. Some of them were provided internet services. Some of them didn't have a computer or a laptop" (Int5)** |
|  |  | **"But you know, there's lots of need around, you know, with intergenerational families sharing technology. So you know, maybe the youth was assigned a computer that or you know, a tablet that did have data on it, but then that youth might have to be sharing it with siblings or the adults" (Int6)** |
|  | **Low Bandwidth and Network Connectivity Issues** | **"But also, we wanted to be mindful of the bandwidth that people have like literal Internet bandwidth. Because we knew that like having lots of different websites to click out to might not work, it's probably really confusing and some of our youth as young as 10" (Int3)** |
|  |  | **"But your mileage may vary some flat lessons, you know, and write in an hour, 45 minutes, or 45 minutes or an hour mark and some and take an hour and a half to take, you know, to happen, which has sometimes to do with like network connectivity issues, because our team sometimes will spend a good amount of time like their internet will cut out or the youth’s internet cut out. And so, then you spend some time with logging back in or like making sure things are working" (Int3)** |
|  |  | **"But it's challenging, you know, as you pointed out, like we have places where the internet is really unreliable, and it's really spotty. And so, I think that's really challenging when you know, the session start and then maybe they drop, and they've got to pick them back up again. And, you know, if that happens too many times, you're that that youth is gonna, they're gonna sign up" (Int4)** |
|  |  | **"Some tribes don't have the broadband that connects certain phone services. For internet, so I know some communities have posted up like hotspot locations for people to use" (Int5)** |
|  |  | **"Yeah, yeah. So it's been a lot of technical support for tribes that have varying capacity and resources. So it really varies from you know, rural like not being able to find hotspots, no IT, multi-generational families within a household to urban, in some of the urban areas like Portland" (Int6)** |
|  |  | **"Yeah, I think overall, we know that youth are able to access and have yet are able, yeah, have technology, resources. And so if that's the case, then this is the best platform to do it. So yeah, and I think it's, you know, it's a pretty small number of folks who have absolutely no IT capacity whatsoever, it seems like most tribes, at least, you know, can lend a laptop and a thumb drive. And it's not a perfect scenario. But that's yeah, an adaptation that folks are making" (Int6)** |
|  |  | **"So there are COVID relief funds, and some of those funds are going out to tribes to, in most tribes that sounds like are able to purchase IT equipment, and either give it to families or either lend it to them, they have to sign out for it" (Int6)** |
|  | **Dealing with Youth Internet Access** | **"One of the things that I've been talking to a few folks about is that, you know, what is the commonality in most programs that I've delivered over again, the last, like 15 years of my career has been that transportation is consistently an issue everywhere you go. So, even if you operate an after-school program, young people, they don't have a ride home can't stay, they've got to catch the bus to get home. Young people in rural communities are often not able to participate in programs because they can't get a ride to wherever the program is happening" (Int1)** |
|  |  | **"And then at least in schools, they can still access the program and complete everything, and they don't have to worry about internet connection or like the bandwidth" (Int4)** |
|  |  | **"Some of them didn't have a computer or a laptop. So they they're currently some of them are renting a laptop from the school just to get through" (Int5)** |
